# Metabolic Reprogramming for Producing Energy and Reducing Power in Fumarate Hydratase Null Cells from Hereditary Leiomyomatosis Renal Cell Carcinoma

**DOI:** 10.1371/journal.pone.0072179

**Published:** 2013-08-15

**Authors:** Youfeng Yang, Andrew N. Lane, Christopher J. Ricketts, Carole Sourbier, Ming-Hui Wei, Brian Shuch, Lisa Pike, Min Wu, Tracey A. Rouault, Laszlo G. Boros, Teresa W.-M. Fan, W. Marston Linehan

**Affiliations:** 1 Urologic Oncology Branch, Center for Cancer Research, National Cancer Institute, National Institutes of Health, Bethesda, Maryland, United States of America; 2 J.G. Brown Cancer Center, University of Louisville, Louisville, Kentucky, United States of America; 3 Center for Regulatory and Environmental Analytical Metabolomics (CREAM), University of Louisville, Louisville, Kentucky, United States of America; 4 Seahorse Bioscience, North Billerica, Massachusetts, United States of America; 5 Molecular Medicine Program, Eunice Kennedy Shriver National Institutes of Child Health and Development, Bethesda, Maryland, United States of America; 6 SIDMAP LLC, Los Angeles, California, United States of America; 7 University of California Los Angeles School of Medicine, Los Angeles, California, United States of America; 8 Department of Chemistry, University of Louisville, Louisville, Kentucky, United States of America; University of South Alabama, United States of America

## Abstract

Fumarate hydratase (FH)-deficient kidney cancer undergoes metabolic remodeling, with changes in mitochondrial respiration, glucose, and glutamine metabolism. These changes represent multiple biochemical adaptations in glucose and fatty acid metabolism that supports malignant proliferation. However, the metabolic linkages between altered mitochondrial function, nucleotide biosynthesis and NADPH production required for proliferation and survival have not been elucidated. To characterize the alterations in glycolysis, the Krebs cycle and the pentose phosphate pathways (PPP) that either generate NADPH (oxidative) or do not (non-oxidative), we utilized [U-^13^C]-glucose, [U-^13^C,^15^N]-glutamine, and [1,2- ^13^C_2_]-glucose tracers with mass spectrometry and NMR detection to track these pathways, and measured the oxygen consumption rate (OCR) and extracellular acidification rate (ECAR) of growing cell lines. This metabolic reprogramming in the FH null cells was compared to cells in which FH has been restored. The FH null cells showed a substantial metabolic reorganization of their intracellular metabolic fluxes to fulfill their high ATP demand, as observed by a high rate of glucose uptake, increased glucose turnover via glycolysis, high production of glucose-derived lactate, and low entry of glucose carbon into the Krebs cycle. Despite the truncation of the Krebs cycle associated with inactivation of fumarate hydratase, there was a small but persistent level of mitochondrial respiration, which was coupled to ATP production from oxidation of glutamine-derived α–ketoglutarate through to fumarate. [1,2- ^13^C_2_]-glucose tracer experiments demonstrated that the oxidative branch of PPP initiated by glucose-6-phosphate dehydrogenase activity is preferentially utilized for ribose production (56-66%) that produces increased amounts of ribose necessary for growth and NADPH. Increased NADPH is required to drive reductive carboxylation of α-ketoglutarate and fatty acid synthesis for rapid proliferation and is essential for defense against increased oxidative stress. This increased NADPH producing PPP activity was shown to be a strong consistent feature in both fumarate hydratase deficient tumors and cell line models.

## Introduction

Hereditary leiomyomatosis and renal cell carcinoma (HLRCC) is an autosomal dominant hereditary cancer syndrome characterized by a predisposition to develop cutaneous and uterine leiomyomas and a very aggressive form of papillary kidney cancer [1–7]. HLRCC-associated renal tumors demonstrate a distinctive architectural and morphology and have a propensity to metastasize early [8]. The predisposition of HLRCC-associated kidney cancer to readily metastasize to both regional and distant lymph nodes is distinctly different and significantly more aggressive than other types of genetically defined kidney cancer.

The primary genetic alteration associated with HLRCC is a germline mutation of the *FH* gene that encodes fumarate hydratase (FH), which is both a tumor suppressor gene and an enzyme of the Krebs cycle [9–11]. Several studies have demonstrated a high *FH* mutation detection rate in HLRCC families and the subsequent loss of the remaining somatic copy in the kidney tumors [12–14].

Mutations of several genes that encode enzymes of the Krebs cycle have recently been implicated in multiple aspects of cancer genetics and progression, and have highlighted the potential importance of altered metabolic states in cancer cells [15–17].

Recently, two HLRCC kidney cancer lines, UOK262 and UOK268, have been established and characterized [18,19]. UOK262 was isolated from a metastatic retroperitoneal lymph node, while UOK268 was isolated from a primary renal lesion in a separate individual. These HLRCC cell lines have been shown to undergo major metabolic transformations; their energy production is derived largely from glycolysis rather than oxidative phosphorylation, and low activity of the master metabolic regulator AMP-dependent kinase (AMPK) reduces p53 levels and activates anabolic factors, such as acetyl CoA carboxylase and rpS6 expression [18,20]. Associated with the absence of FH enzymic activity, and thus the loss of a complete Krebs cycle, glutamine provides carbon for fatty acid biosynthesis by reductive carboxylation of glutamine-derived α-ketoglutarate [21], and contributes to a high accumulation of fumarate [22].

The mitochondrial defects and high fumarate accumulation, as a result of fumarate hydratase dysfunction, has several important consequences for the cancer cell. First, high mitochondrial concentrations of fumarate may cross into the cytosol via dicarboxylate carriers to inhibit the activity of the prolyl hydroxylases, which target HIF-1α for VHL-dependent degradation under normoxia, thus rendering HIF-1α constitutively active [23]. Second, the high levels of fumarate can result in a large degree of non-specific succination of cysteine residues of proteins. The consequences of such protein modification have yet to be fully elucidated but could include the release of Nrf2 from succinated KEAP1 resulting in a beneficial up-regulation of the antioxidant response pathway [24,25]. Furthermore, UOK262 cells generate superoxide at a high rate and have constitutively elevated levels of superoxide and peroxide (ROS), which is another important mediator of HIF-1α stabilization [26,27].

These metabolic adaptations only partly account for the cell’s ability to continue to survive and proliferate. It is assumed that the impaired mitochondria are unable to produce ATP by respiration-coupled oxidative phosphorylation, due to the inability of substrates to move completely through the Krebs cycle, and therefore cells must rely mainly on glycolysis to maintain the ATP levels. In addition, increased proliferation requires up-regulation of RNA and DNA biosynthesis, which is tightly coupled to the cell cycle and requires the input of the glycolytic, Krebs cycle and pentose phosphate pathway (PPP) metabolites [28–30]. Moreover, both reductive carboxylation and defense against ROS require NADPH.

The main potential sources of cytoplasmic NADPH include isocitrate decarboxylation catalyzed by NADP^+^-dependent IDH1, the activity of malic enzyme 1, and the oxidative branch of the PPP. However in FH null cells, the IDH1/2-catalyzed reaction may be a net consumer of NADPH due to its use in reductive carboxylation [21]. The most efficient source of NADPH is the oxidative branch of PPP, which produces two NADPH from oxidation of one glucose molecule to ribose. Although some cancers mainly utilize the oxidative PPP branch [31], like normal, non-transformed cells, other cancers, with a fully functional Krebs cycles, heavily utilize (>85%) the non-oxidative PPP for ribose biosynthesis [32]. {Centelles, 2007 #628; Boros, 1997 #448; Centelles, 2007 #628}It has been proposed that the balance between these branches may be important in carcinogenesis [33].

Here we report the metabolic adaptations involved in NADPH production and nucleotide metabolism in the FH (-/-) patient-derived UOK262 and UOK268 cell lines. In addition, we have produced UOK262 cells containing a stable transfection of wild-type fumarate hydratase (UOK262WT) or with an empty vector (UOK262EV). Unlike UOK262, UOK262WT has been previously shown not to be tumorigenic, as it fails to produce tumors in nude mice [20]. The metabolic reprogramming was interrogated using a systems biochemistry approach by coupling ^13^C tracers with mass spectrometry [34] and NMR [35,36] to determine which adaptations in these cells generate anabolic precursors for nucleotide biosynthesis, metabolic energy for proliferation, and NADPH for fatty acid biosynthesis and removal of ROS. We further used simultaneous measurements of oxygen consumption and extracellular acidification [37] to determine the extent of respiration-coupled oxidative phosphorylation and glycolysis of these cells. We showed that the cells retain and increase their usage of the oxidative branch of the PPP, generating NADPH for necessary cellular functions, and that the residual oxygen consumption associated with oxidation of glutamine to fumarate in the UOK262 cells remained coupled to ATP production.

## Results

### Respiration rates and oxidative phosphorylation are low but significant in FH null cells

**Figure 1:**
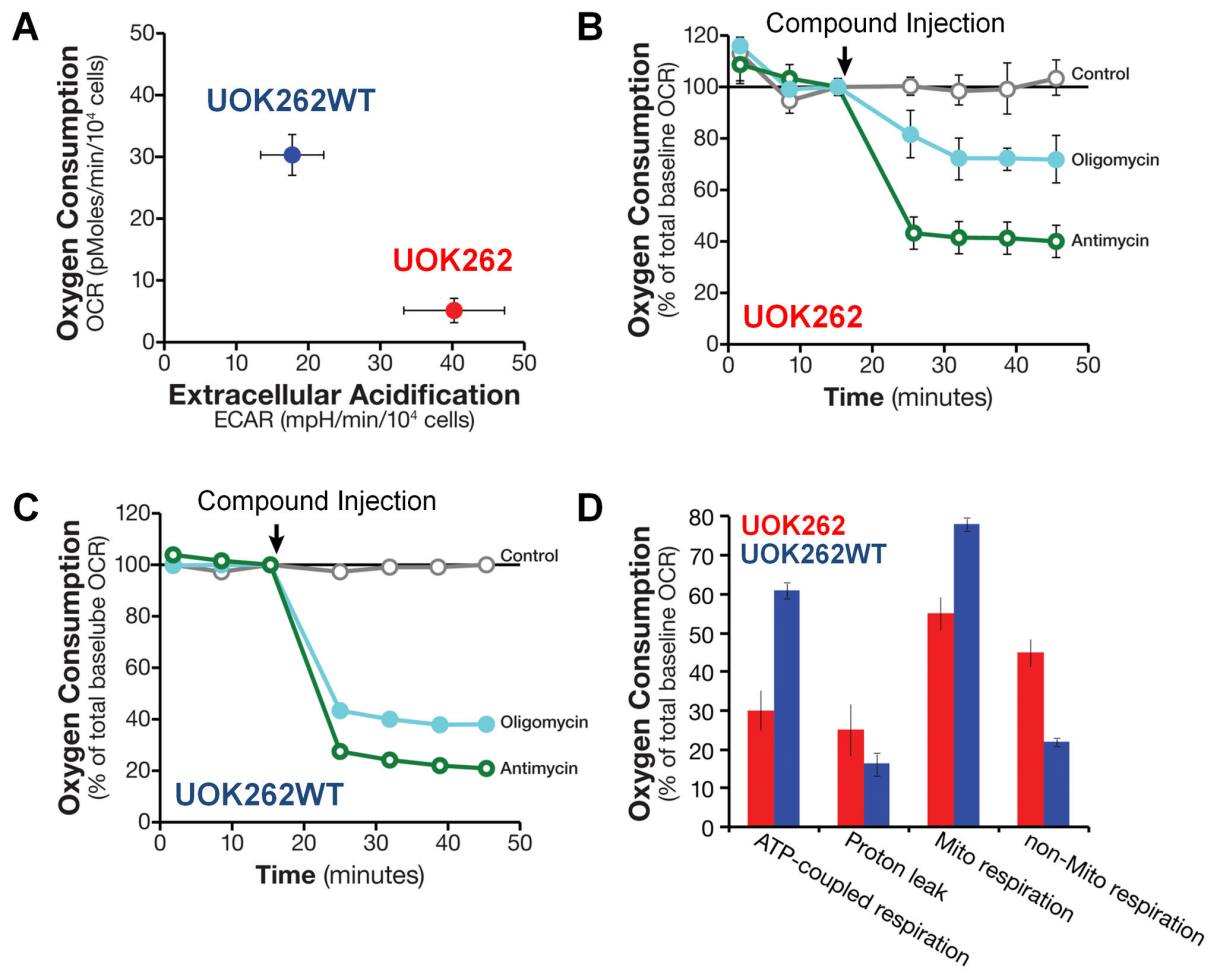
Mitochondrial and ATP-coupled oxygen consumption rates (OCR) were lower but extracellular acidification rates (ECAR) were higher in UOK262 than UOK262WT cells. Oxygen consumption rates (OCR) and extracellular acidification rates (ECAR) were determined using the Seahorse XF96 Analyzer in cells as described in Methods:. **A** - OCR versus ECAR for UOK262 and UOK262WT cells normalized to 10^4^ cells. **B** & **C** - Inhibition of the mitochondrial ETC and oxidative phosphorylation in UOK262 (**B**) and UOK262WT cells (**C**) using respectively Antimycin (1 µM) and Oligomycin (1 µM), which were added to the cells after the basal OCR was established (as indicated by arrow). The data are expressed as % of basal OCR. **D** - Comparison of ATP-coupled respiration, proton-leak, mitochondrial respiration, and non-mitochondrial respiration of UOK262 and UOK262WT cells, which were calculated from data in Panels A-C. All error bars represent standard deviation.

To investigate and quantify aerobic glycolysis and mitochondrial respiration in FH null kidney cancer cells, measurements were made of the oxygen consumption (OCR) and extracellular acidification rates (ECAR), along with tracing of the carbon flow from glucose to lactate and from glutamine to fumarate. Figure 1A shows the oxygen consumption versus extracellular acidification in UOK262 and UOK262WT cells. UOK262 cells have a low OCR and a high ECAR, whereas UOK262WT cells have a much higher (4-fold) OCR and a two-fold lower ECAR (Table 1), which supports at least a partial reversion to a wild type FH phenotype. Additionally, fumarate hydratase enzyme activity assays demonstrated the absence of activity in UOK262 and UOK262EV, but recovery of 63.2% of the fumarate hydratase activity in UOK262WT when compared to the HRCE control cells (Figure S1 in File S1). The control UOK262EV had OCR and ECAR levels similar to that of UOK262, while in the non-metastatic UOK268 line the OCR was low, but slightly higher than UOK262 line, and the ECAR was high, but slightly lower than that of UOK262 (data not shown [19]). Low oxygen consumption and high lactate production is expected for cells with a defective fumarate hydratase that interrupts the Krebs cycle, rendering them more reliant on glycolysis for ATP production.

**Table 1:** Oxygen consumption and extracellular acidification rates in FH null and restored cells.

**Cell Line**	**OCR (pmoles/min/10^4^ cells)**	**ECAR (pmoles/min/10^4^ cells)**
**UOK262**	7.2 ± 1.6	40.9 ± 7.4
**UOK262WT**	29.87 ± 2.5	17.8 ± 4.4
**Ratio 262WT/262**	4.13 ± 1.75	0.43 ± 0.19

OCR: oxygen consumption rate ECAR: extracellular acidification rate

Further investigation of the OCR in UOK262 cells demonstrated that low level mitochondrial respiration still persisted, as shown by an approximate 60% decrease in the UOK262 baseline OCR by antimycin, a complex III inhibitor (Figure 1B). Results of the antimycin experiments suggest that the remaining 40% of the basal OCR was of non-mitochondrial origin, potentially being used in processes such as the oxidative branch of the pentose phosphate pathway (Figure 1B and D). Furthermore, the mitochondrial respiration was coupled to oxidative phosphorylation, as shown by a n approximate 30% decrease in basal OCR by oligomycin, an inhibitor of ATP synthase (Figure 1B). This suggests that ~50% of the residual mitochondrial respiration of the UOK cells was coupled to ATP production. As the remaining 50% of the OCR was inhibited neither by antimycin nor by oligomycin, this most likely represents proton leakage (non-ATP producing futile cycle of respiration) that would contribute to oxidative stress. In contrast, the ECAR was not affected by any of the mitochondrial inhibitors, indicating that glycolysis in UOK262 was already at its maximal capacity (data not shown). Restoring wild type fumarate hydratase in UOK262 cells raised the baseline OCR and increased mitochondrial respiration and ATP-coupled respiration and reduced proton leakage (Figure 1C & 1D).

**Figure 2:**
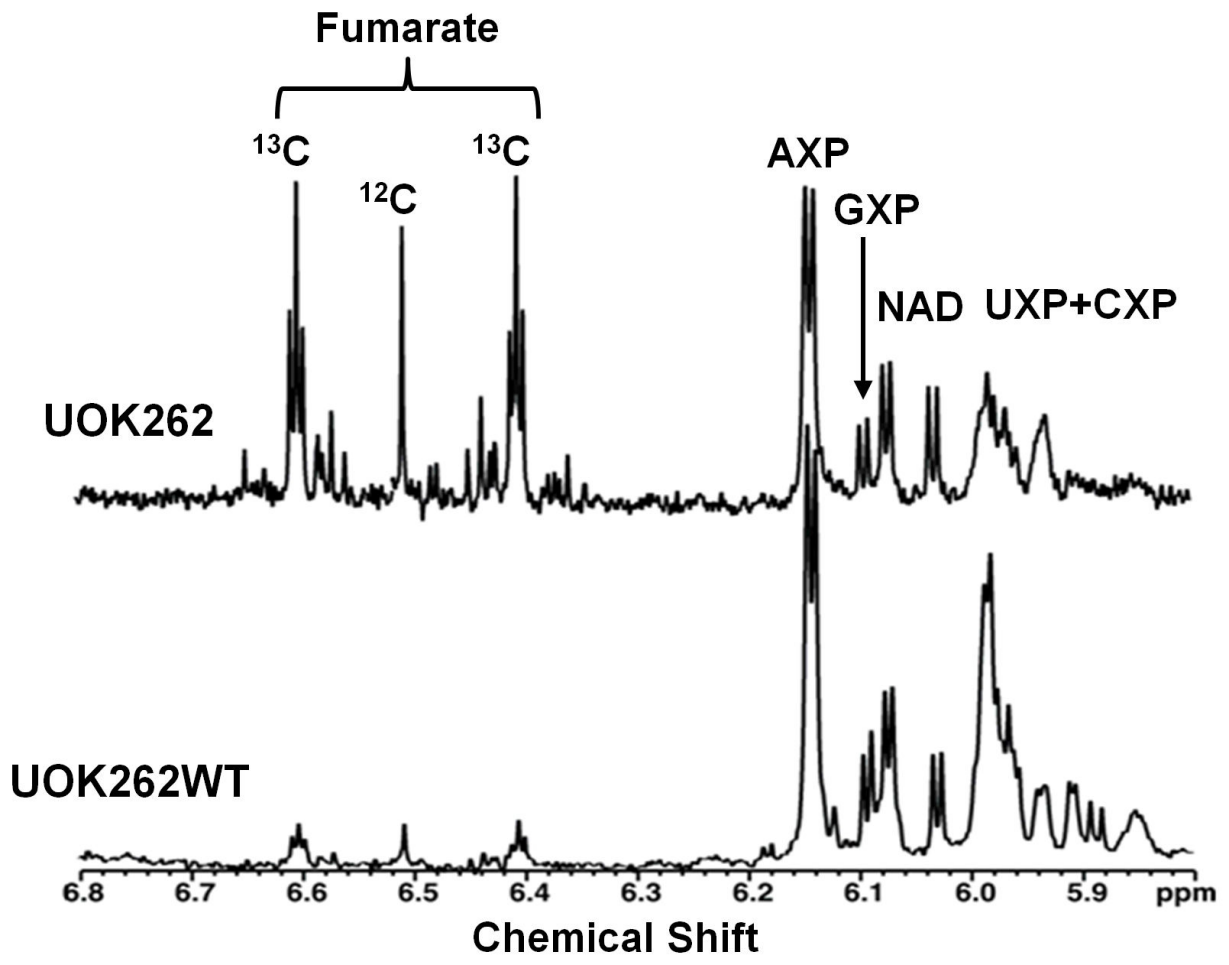
^1^H NMR spectra show higher fumarate accumulation and ^13^C enrichment from glutamine in FH null than FH restored cells. 1D ^1^H NMR spectra were recorded at 800 MHz, 20 °C with an acquisition time of 2 s and a recycle time of 5 s. The data were processed using an unshifted gaussian and a 1 Hz line broadening exponential function. Both UOK262 and UOK262WT were cultured in 10 mM ^12^C glucose and 4 mM [U-^13^C,^15^N]-glutamine for 24 h before measurements were made. Note the dual strong ^13^C satellites in the fumarate resonance at 6.64 and 6.37 ppm, showing that the fumarate is derived readily from glutamine in UOK262 cells but less so in UOK262WT cells. The triplet structure of the satellites results from long range coupling to the neighboring ^13^C, which is consistent with the presence of ^13^C_4_-fumarate as a product of glutamine oxidation via the Krebs cycle [2]. In contrast, nucleotide ribose resonances (H1’) show no ^13^C satellites, indicating that glutamine is not a significant carbon source for ribose biosynthesis in both cell types.

Our results from HLRCC derived human tumor cell lines agree with the previous report on the effect of FH ablation in immortalized mouse kidney cells [22], where glutamine is oxidized through part of the Krebs cycle to produce NADH/FADH_2_ and high levels of fumarate. We have directly confirmed the dominance of glutamine conversion to fumarate in UOK262 cells using [U-^13^C]-glutamine as tracer (Figure 2). In contrast, little fumarate was derived from glucose in these FH null cells grown with [U-^13^C]-glucose tracers (Figure S2A in File S1). Interestingly, in the UOK262WT (FH restored) cells, although the fumarate production was significantly decreased it was still derived mainly from glutamine rather than glucose (Figure 2 and Figure S2A in File S1). This glutaminolytic pathway requires several Krebs cycle enzymes to convert glutamine via α-ketoglutarate to fumarate to generate one NADH, one FADH_2_ and one GTP. Re-oxidation of NADH and FADH_2_ by oxygen via the electron transport chain may generate up to 4 ATP molecules, which is consistent with the observed ATP-coupled oxygen consumption by UOK262 (Figure 1B). Such truncated oxidative phosphorylation generates less ATP than normal oxidative phosphorylation achieved by complete oxidation of pyruvate to 3 CO_2_ (which can generate up to 12.5 ATP equivalents). This also accounts in large measure for the decreased mitochondrial OCR in UOK262 cells relative to UOK262WT cells (Figure 1A).

### FH null cells manifest enhanced aerobic glycolysis

Generating energy by accelerated lactic fermentation under aerobic conditions, as opposed to oxidative phosphorylation, is the hallmark of the “Warburg effect”. By following ^13^C atoms from labeled glucose into lactate, it is possible to determine both the lactic acid fermentation capacity and the fraction of lactate derived from consumed glucose [2,36].

Using [U-^13^C]-glucose tracing in both the FH inactive and FH restored cell lines, we compared labeled glucose consumption and the release of the primary glycolytic product, ^13^C_3_-lactate, from which the fraction of glucose converted to lactate was determined. While the rate of glucose consumption over 24 h was slightly higher in the UOK262 cells than that in UOK262WT (FH restored), the rate of ^13^C_3_-lactate release increased substantially (Figure S2B in File S1), accounting for the doubling in the fraction of glucose conversion to excreted lactate in UOK262 cells (Table 2). This agrees with the previous non tracer-based estimates in mouse kidney cells, where the fraction of excreted lactate increased from about 30% in the parent cells to more than 80% in the FH knockout cells [22]. Lactate excretion was also accompanied by extracellular acidification and ECAR is known to correlate with the glycolytic activity [37]. Our measured ECAR was approximately twice as high in the UOK262 line compared with the FH restored UOK262WT (Figure 1A, Table 1), which suggests that extracellular acidification is mainly the result of the proton symport with excretion of glucose-derived lactate.

**Table 2:** Conversion of [U-^13^C]-glucose to extracellular ^13^C_3_-lactate in FH null and restored cells.

**Cell Line**	**Δ[Glc]/24h^a^**	**Δ[Lac]/24 h^b^**	**0.5 ΔLac/ΔGlc^c^**	**%^13^C in Lac^d^**
**UOK262**	3.9 ± 0.2	5.2 ± 0.5	0.67 ± 0.1	96 ± 2
**UOK262WT**	3.7 ± 0.1	2.3 ± 0.1	0.31 ± 0.02	96 ± 2

Values were determined by NMR as described in the text.

a. consumption of glucose in 24 h

b. production of ^13^C lactate in 24 h

c. Fractional glucose converted to lactate

d. % enrichment of extracellular lactate from [U-^13^C]-glucose

We noted that a large fraction of the intracellular lactate and alanine were present as the ^13^C_3_ form in both UOK262 and UOK262WT cells (Figure S3A in File S1; Table S1 in File S1), which is consistent with their production from [U-^13^C]-glucose via glycolysis. Additionally, the [U-^13^C]-glutamine tracing did not detect incorporation of glutamine-derived carbon into lactate or alanine (Table S1 in File S1, Figure S3 in File S1) for either cell line. These findings are consistent with the findings in other cancer cell lines where glutamine contributes little to lactate production, compared with glucose [2,38,39].

**Figure 3:**
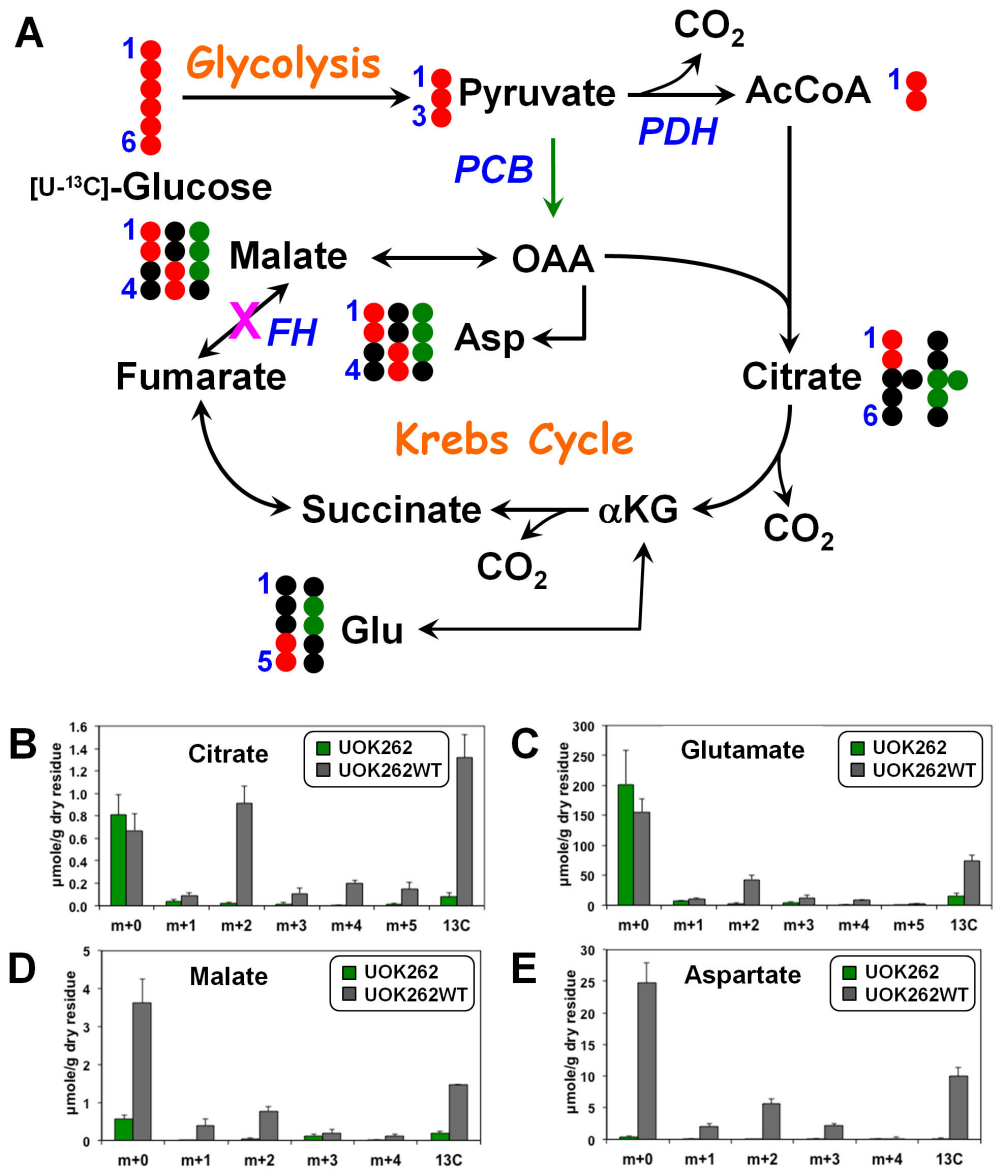
Levels and patterns of ^13^C incorporation from [U-^13^C]-glucose into Krebs cycle metabolites indicate that the Krebs cycle is blocked in FH null cells but functional in FH restored cells. Panel A illustrates the fate of glucose ^13^C atoms in various glycolytic and Krebs cycle metabolites. The Krebs cycle block at FH in UOK262 cells is indicated by a pink **X**. The black circles represent ^12^C, whereas the red and green circles represent ^13^C incorporated via the Krebs cycle without and with pyruvate carboxylation input, respectively [2,3]. Single- and double-headed arrows show irreversible and reversible single step reactions while dashed arrows indicate multiple step reactions. PDH: pyruvate dehydrogenase; PCB: pyruvate carboxylase; FH: fumarate hydratase; AcCoA: acetyl CoA; OAA: oxaloacetate; αKG: α−ketoglutarate. For the four Krebs cycle metabolites, UOK262WT cells show much higher levels of ^13^C incorporation than the parent UOK262 cells, which is consistent with the restoration of the FH and thus full Krebs cycle activity. UOK262 and UOK262WT cells were grown in [U-^13^C]-glucose for 24 h before extraction. The Krebs cycle metabolites, citrate (B), glutamate (C), malate (D) and aspartate (E) in the cell extracts were analyzed by GC-MS for levels (µmole/g dry residue) of singly (M+1) to quintuply (M+5) ^13^C labeled isotopologues, as described in Methods. Also shown is the total level of all isotopologues for each metabolite. Error bars represent the standard error of the mean (SEM).

Moreover, approximately one third of the glucose consumed by UOK262 cells was used for metabolic purposes other than glycolysis (Table 2), which could include the production of nucleic acids (e.g. the ribose moiety) and production of citrate and oxidation to CO_2_ via the Krebs cycle. The incorporation of the ^13^C atoms from [U-^13^C]-glucose into key Krebs cycle components (citrate and malate) and derivatives (aspartate and glutamate), either via acetyl CoA via citrate synthase or directly from pyruvate via pyruvate carboxylase (PCB), was compared between UOK262 and UOK262WT (Figure 3A). In UOK262WT cells, these four marker metabolites showed more of the m+2 than the m+3 isotopologue (Figure 3B–E), indicating that the glucose carbon entered mainly via citrate synthase, but with a significant component from the anaplerotic pyruvate carboxylase route. In contrast, not only were the total levels of these metabolites lower than in the WT cells, but also the degree of labeling was very small, indicating a greatly diminished Krebs cycle, and very little incorporation of glucose carbon (Figure 3B–E). Nevertheless, a small m+3 peak was observed in the UOK262 the ^13^C atoms were detected in, that probably represents pyruvate carboxylase (PCB) activity.

Altogether, these glucose tracer data indicate that FH dysfunction leads to enhanced glycolysis or the “Warburg effect” that can be reversed by the re-introduction of FH function without additional changes.

### FH null cells primarily use and maintain the oxidative branch of the pentose phosphate pathway for ribose and NADPH production

**Figure 4:**
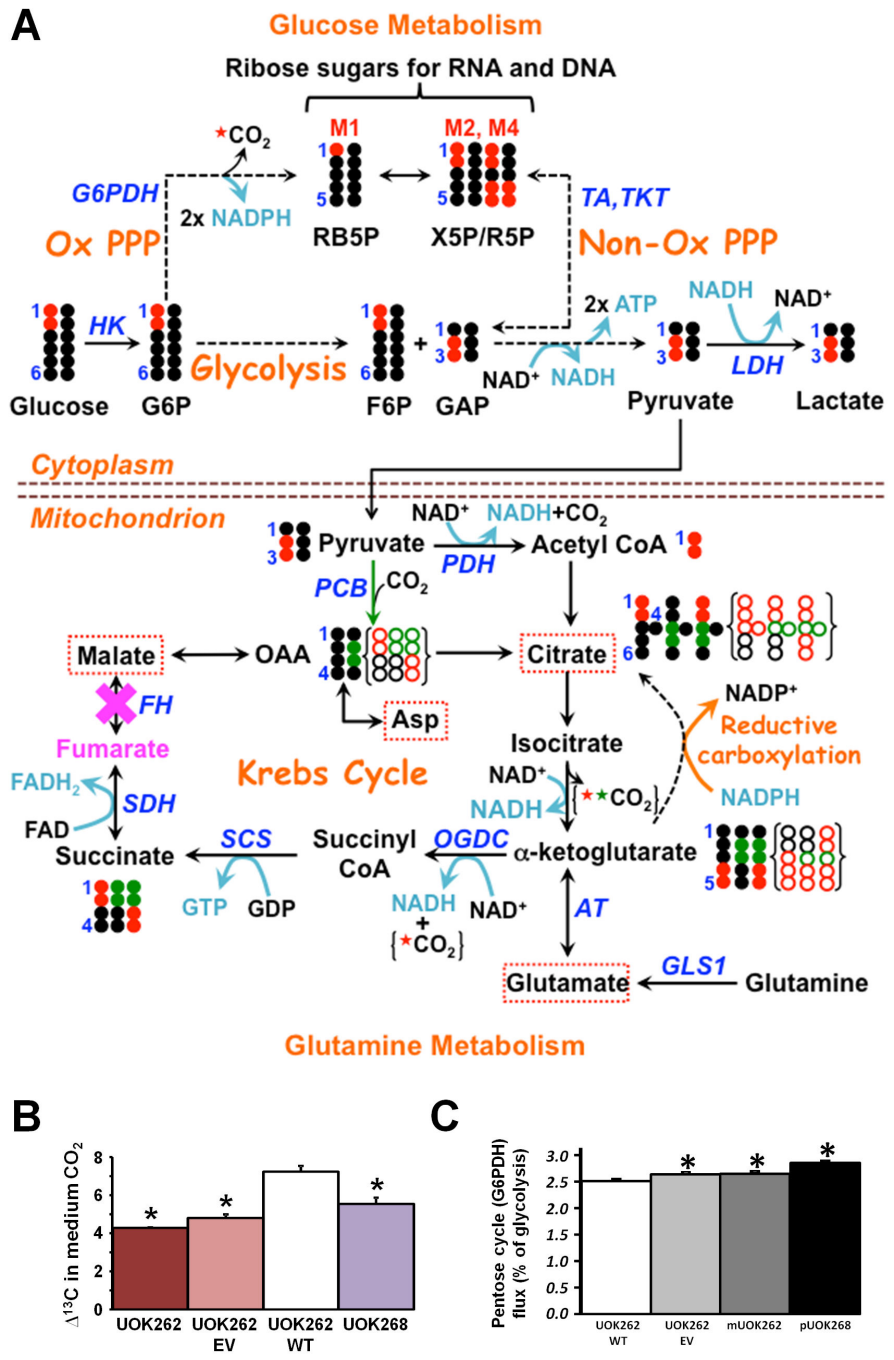
^13^C tracing of glucose and glutamine metabolism that delineates the production and/or labeling patterns of pentoses, reducing and energy equivalents, and CO_2_ for FH null and restored cells. Panel **A** shows the atom-resolved fate of 50% [1,2- ^13^C_2_]-glucose plus 50% all ^12^C glucose through glycolysis and the pentose phosphate pathway (PPP, both oxidative and non-oxidative branches). Also shown is the expected [1,2- ^13^C_2_]-glucose and glutamine oxidation in FH null (UOK262) or restored (UOK262WT) cells via glycolysis, PPP, and/or the Krebs cycle to produce energy (ATP and GTP), reducing equivalents (NADH, NADPH, and FADH_2_) and ^13^CO_2_ (from C1 of glucose, C4 of citrate and C1 of α-ketoglutarate). The reoxidation of NADH and FAHD2 via the ETC can produce up to 5 ATP while 2 ATP are generated per glucose oxidation via glycolysis. The ^13^C labeling patterns become scrambled after the SCS step due to the molecular symmetry of succinate but the scrambled patterns are not shown. The consumption of NADPH by the reductive carboxylation of α-ketoglutarate to form citrate is noted [21]. Moreover, Metabolites marked by red dashed rectangles had their ^13^C labeling patterns derived from [U-^13^C]-glucose shown in Figure 4A–D. The black circles represent ^12^C, while the red circles or stars and the green circles or stars represent ^13^C derived from the first turn of the Krebs cycle respectively without and with pyruvate carboxylation input; the circles with holes in them enclosed in brackets depict the corresponding ^12^C and ^13^C fate during the second turn of the Krebs cycle (note that ^13^CO_2_ can only be liberated from [1,2- ^13^C_2_]-glucose starting with the second turn); blockade at FH is noted by a pink **X**, resulting in fumarate accumulation (cf. Figures 3 & S1A); M1, M2, and M4 refer to the singly, doubly, and quadruply ^13^C labeled isotopologues; the enzymes are denoted in blue italics; solid and dashed arrows represent single and multi-step reactions, respectively; single- and double-headed arrows depict irreversible and reversible reactions, respectively. G6P: glucose 6-phosphate; HK: hexokinase; G6PDH: glucose-6-phosphate dehydrogenase; RB5P: ribulose-5-phosphate; X5P/R5P: xylulose/ribose-5-phosphate; F6P: fructose 6-phosphate; GAP: glyceraldehyde-3-phosphate; TKT: transketolase; TA: transaldolase; LDH: lactate dehydrogenase; PDH: pyruvate dehydrogenase; PCB: pyruvate carboxylase; OAA: oxaloacetate; GLS1: glutaminase 1; AT: aminotransferases; OGDC: 2-oxoglutarate decarboxylase; SCS: succinyl CoA synthetase; SDH: succinate dehydrogenase; FH: fumarate hydratase. Panel **B** shows the relative level of flux through the glucose-6-phosphate dehydrogenase (G6PDH) enzyme of the oxidative branch of the PPP as determined by Gas chromatography/mass spectrometry (GC/MS) of UOK262, UOK262EV, UOK268 and UOK262WT. This was measured based on glucose consumption as mmol/hour per g tissue as previously described [50] and presented as a percentage of the flux in UOK262WT. The bar graph shows the mean values of three replicates along with the standard deviation as error bars. * indicates a statistically significant result (p<0.05) from a t-test. Panel **C** shows the lower ^13^C enrichment in CO_2_ dissolved in the culture media of the three FH null cells than in that of the FH restored cell. This is consistent with the inability of the FH null cells to oxidize glucose in the second turn of the Krebs cycle due the blockade at FH, which leads to no ^13^CO_2_ release from glucose oxidation via the Krebs cycle. As such, it is reasonable to assume that a large fraction of the ^13^CO_2_ generated from [1,2- ^13^C_2_]-glucose oxidation by FH null cells is mediated by the oxidative PPP. For the FH restored cells, both Krebs cycle and oxidative PPP contribute to ^13^CO_2_ release. Δ^13^C was calculated as the amount of ^13^CO_2_ divided by (1000 times the total amount of CO_2_). The bar graph shows the mean values of three replicates along with the standard deviation as error bars. * indicates a statistically significant result (p<0.05) from a t-test.

Other than energy production, an important use of glucose is for the production of ribose sugars via the oxidative or non-oxidative branches of the pentose phosphate pathway (PPP). Tracing with [U-^13^C_2_] glucose demonstrated that glucose is the main source of carbon for ribose synthesis in UOK262 cells (Figure S4 in File S1). Tracing with [1,2- ^13^C_2_] glucose permits the differentiation of ribose sugars produced by the oxidative or non-oxidative branches of PPP, as while ribose produced by the oxidative branch of PPP loses the 1-^13^C carbon of labeled glucose to produce singly labeled ribose (M1), ribose produced by the non-oxidative PPP branch retains both labeled carbons (M2) (Figure 4A) [40].

The analysis was performed on the two FH null parental lines, UOK262 and UOK268, and the isogenic UOK262EV where the oxidative branch accounted for 56-66% of the PPP, as well as the UOK262WT cell line where the oxidative branch accounted for 50% of the PPP (Table 3).

**Table 3:** Fractional distribution of RNA Ribose Isotopologues derived from [1,2- ^13^C_2_]-glucose.

	**^13^C Distribution in RNA Ribose (%)**			
**Cell Line**	**UOK262WT**	**UOK262EV**	**UOK262**	**UOK268**
**M1 Labeled Ribose**	42.4 ± 1.3	47.5 ± 1.7	47.4 ± 1.9	59.5 ± 1.6
**M2 Labeled Ribose**	41.6 ± 1.2	37.9 ± 1.3	38.0 ± 1.5	30.8 ± 1.0
**Total Labeled Ribose**	36.3 ± 1.1	35.4 ± 1.2	45.3 ± 1.4	32.0 ± 1.1
**Oxidative PPP (%) M1/[(M1)+(M2)]**	50.5 ± 1.2	55.6 ± 1.5	55.5 ± 1.7	65.9 ± 1.2

This oxidative branch activity is elevated in comparison to many cancer cell lines, where the oxidative branch is typically reduced and accounts for <20% of the carbon flow through PPP [31,32,41]. Thus, with the increased level of glycolysis, the flux through glucose-6-phosphate dehydrogenase (G6PDH) of the oxidative branch of the PPP is significantly increased in the FH null cell lines compared to the FH restored line producing a significantly increased amount of NADPH per cell (Figure 4B), which can be used to drive lipid biosynthesis and for glutathione synthesis for detoxification of ROS. Moreover, the absolute levels of RNA ribose were higher in UOK262 (24.5%), UOK262EV (13.2%) and UOK268 cells (16.9%) compared to the restored UOK262WT cells (Figure S5 in File S1) consistent with the greater demand for RNA/DNA biosynthesis in rapidly proliferating cells.

An additional feature of the [1,2- ^13^C_2_]-glucose tracer studies is that no ^13^CO_2_ should be liberated during the first turn of the Krebs cycle and only subsequent cycles. As complete cycles require fumarate hydratase activity, only WT cells should produce any ^13^CO_2_ in the Krebs cycle (Figure 4A). Analysis of levels of ^13^CO_2_ in the culture media from the four cell lines indicated that FH null cells (UOK262, UOK262EV, and UOK268) liberated significantly less ^13^CO_2_ than the FH restored UOK262WT cells (Figure 4C). That the levels did not drop further is because ^13^CO_2_ could be generated by other processes, including the oxidative branch of the PPP.

**Figure 5:**
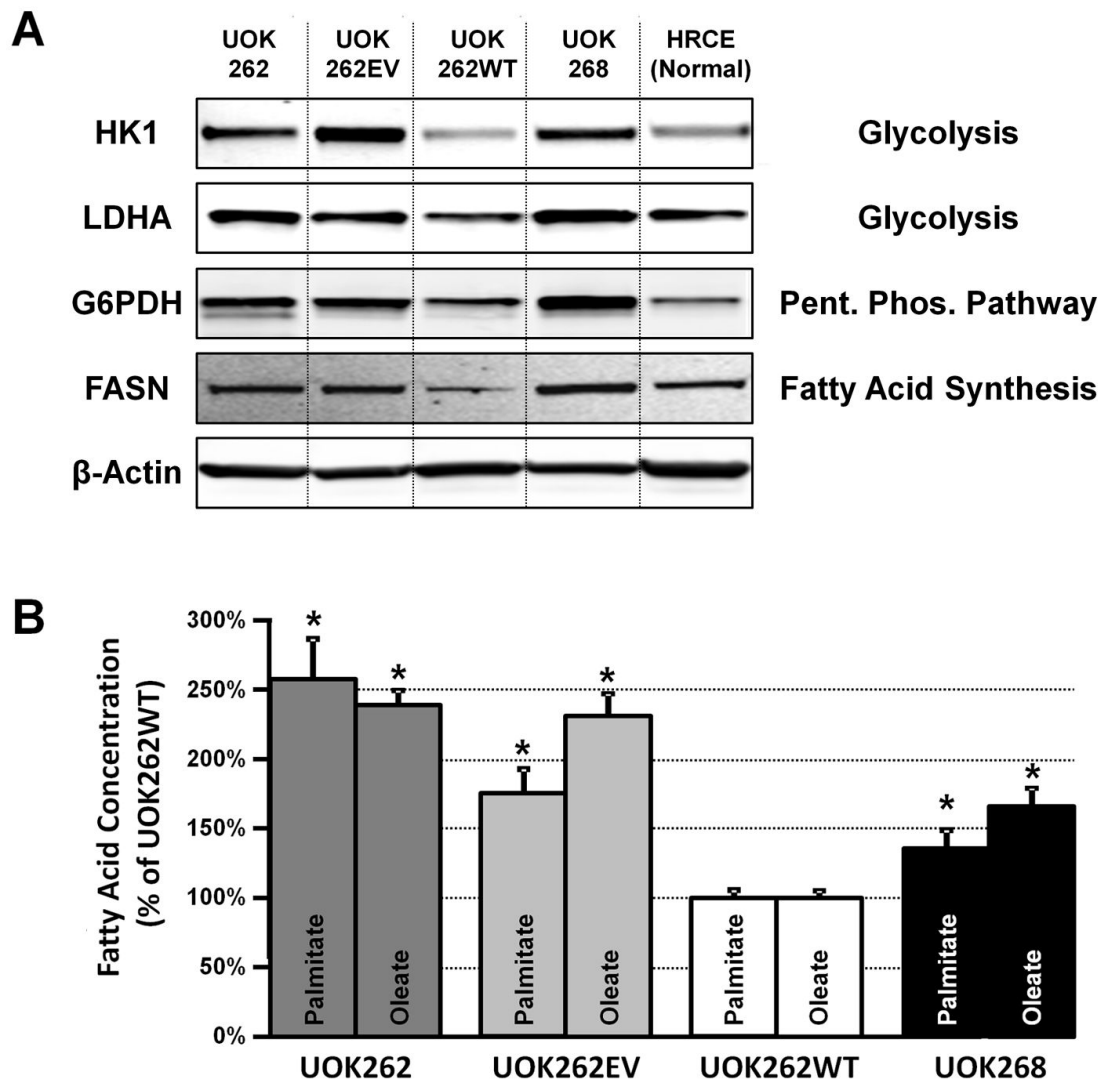
FH null cells have a higher protein expression of Glucose 6-Phosphate Dehydrogenase (G6PDH) than FH restored and normal renal cells. **A** - Western blots for HK1, LDHA, G6PDH, FASN and β-Actin were performed for UOK262, UOK262EV, UOK268 (FH null), UOK262WT (FH restored), and HRCE (human renal cortical epithelial) cells (a representative normal kidney cell), as described in Methods. **B** - The relative levels of two fatty acids (palmitate and oleate) were determined by Gas chromatography/mass spectrometry (GC/MS) of UOK262, UOK262EV, UOK268 and UOK262WT. The bar graph shows the mean values of three replicates along with the standard deviation as error bars. * indicates a statistically significant result (p<0.05) from a t-test.

To corroborate the higher capacity of oxidative PPP in FH null cells Western blot analysis was used determine the protein level of glucose-6-phosphate dehydrogenase (G6PDH), the key enzyme for the oxidative branch of the PPP, in these cells relative to normal human renal cortical epithelial cells (HRCE) (Figure 5A). The level of G6PDH was on average two to threefold higher in UOK262, UOK262EV and UOK268 cancer cells than in the HRCE cells, while FH restoration in UOK262WT cells caused a reduction of the G6PDH level, more closely approximating those observed in the HRCE cells (Figure 5A). When compared with HRCE cells, restoration of FH caused a decrease in expression levels of Hexokinase 1 (HK1) and Lactate Dehydrogenase A (LDHA), two key enzymes involved in lactic fermentation, and for Fatty Acid Synthase (FASN), respectively.

A major use of NADPH in the cell is in the production of fatty acids from acetyl CoA. The levels of two abundant fatty acids, palmitate and oleate, were significantly higher in UOK262, UOK262EV and UOK268 cells compared with UOK262WT cells (Figure 5B), consistent with an increased rate of utilization of NADPH. This requires an increase in the rate of regeneration of NADPH to maintain its level.

**Figure 6:**
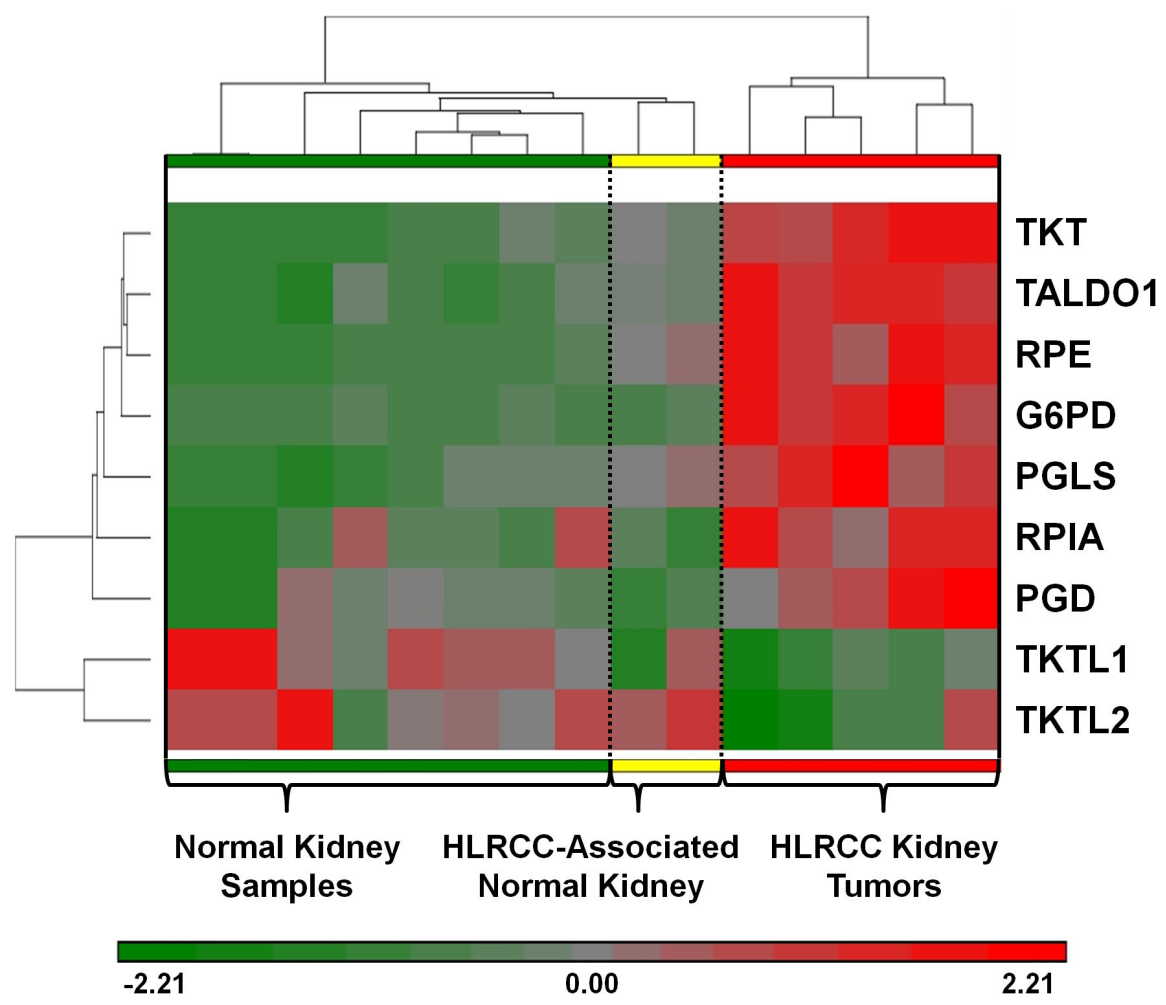
Hierarchical clustering of HLRCC renal tumors and normal kidney based on the expression levels of ribose sugar metabolism genes. The Affymetrix Human Genome U133 Plus 2.0 Array expression data for 5 HLRCC renal cancer samples (red), 2 HLRCC-associated normal kidney samples (yellow) and 8 normal kidney samples (green) [42] was clustered using Euclidean hierarchical clustering (Partek Genomic Suite 6.6) based on the expression data for selected ribose sugar metabolism genes. The heat map was shaded to indicate comparative over-expression in red and under-expression in green. TKT: Transketolase, TALDO1 Transaldolase 1, RPE: Ribulose-5-Phosphate-3-Epimerase, G6PD: Glucose-6-Phosphate Dehydrogenase, PGLS: 6-Phospho-gluconolactonase, RPIA: Ribose 5-Phosphate Isomerase A, PGD: Phosphogluconate Dehydrogenase, TKTL1: Transketolase-like 1, TKTL2: Transketolase-like 2.

Previous work by Ooi et al. had provided Affymetrix Human Genome U133 Plus 2.0 Array expression data for 5 HLRCC renal tumors, 2 HLRCC-associated normal kidney samples and 8 normal kidney samples [42]. An analysis of these data followed by the selection of genes associated with ribose sugar metabolism demonstrated substantial levels of over expression for the selected genes specifically within the HLRCC tumors compared to either their associated normal kidney cells or the normal kidney samples (Figure 6). The array data from tumor samples underscores of the importance of ribose sugar metabolism in HLRCC tumors and implies that the HLRCC cell lines accurately recapitulate the metabolic features of natural HLRCC tumors. However, it is possible that other papillary RCC tumors could have a similar expression profile and that this is not specific to HLRCC. To address this, additional data from Ooi et al. for 22 Papillary Type I and 12 Papillary type II renal tumors was compared to the HLRCC samples for this gene selection. This demonstrated a similar expression profile in only 4 out of 12 Papillary Type II renal tumors, and no such profile was found in Papillary Type I renal tumors (Figure S6 in File S1). This suggests that although a subset of Papillary Type II tumors may share a similar ribose sugar metabolism with HLRCC, it is not a universal observation in papillary renal tumors.

## Discussion

The ECAR and stable isotope tracer experiments show that UOK262 FH null cells are highly glycolytic, with a dominant fraction of glucose consumed metabolized and excreted as lactate (Tables 1 and 2). Despite the blockade of the Krebs cycle at the FH step, UOK262 FH -/- cells continue to respire, albeit at a low rate, compared with the UOK262WT FH restored cells (Figure 1A). Although, in UOK262 FH -/- cells there is a greater degree of non-mitochondrial oxygen usage and only about half of the residual mitochondrial respiration is still coupled to oxidative phosphorylation (Figure 1B & 1D). This is consistent with the concept that more of the UOK262 oxygen usage would be related to the oxidative branch of the pentose phosphate pathway (non-mitochondrial) and that previously observed increases in reactive oxygen species (ROS) would be related to the increased proton leakage from reduced coupling [26,27]. It is clear from this and previous reports [21] that the primary fuel for this residual oxidative phosphorylation is glutamine, which ends up as fumarate and to a lesser extent as heme [22] with concomitant production of 1 CO_2_ and up to 5 ATP equivalents per glutamine converted to fumarate. When FH is restored in UOK262WT cells, the decreased glucose consumption and lactic fermentation rates, the 4-fold higher oxygen consumption rate (Figure 1A), doubling of ATP-coupled respiration (Figure 1D) and gain of fumarate hydratase activity (Figure S1 in File S1), including within the mitochondria, indicate a restoration of oxidative phosphorylation and a diminished reliance on glycolytic ATP production. Thus, the restoration of FH leads towards a more normal metabolic phenotype, rather than a lactic fermentation driven “Warburg”-like phenotype, with increased usage of mitochondrial respiration and loss of extreme glucose dependence.

It is also notable that although the Krebs cycle is truncated at fumarate in UOK262 cells, the large accumulation of glutamine-derived fumarate implies that continued production of fumarate could be important to these cells. Firstly, it provides a steady source of fumarate to replace that lost to the environment and used for the succination of newly synthesized proteins and secondly, it implies that succinate dehydrogenase (complex II) remains functional, which is important for proliferating cells. A critical step in *de novo* pyrimidine biosynthesis is the action of dihydroorotate dehydrogenase (DHODH) a mitochondrial enzyme that requires complex II activity. The sustained DHODH activity in UOK262 cells should help meet their high demand for pyrimidine biosynthesis. Proliferation requires active RNA metabolism; these cells are highly proliferative, and have a higher total RNA content.

The high demand for nucleotides in FH null cells also requires a supply of ribose, which we have shown in these cells to be produced in larger amounts from glucose via increased flux through the PPP. Notably, the UOK262WT FH-restored cells still retained a high degree of ribose production via the oxidative PPP pathway for ribose production (Table 3), indicating either this is characteristic of normal kidney epithelial tissue or retention of some residual metabolic reprogramming. This second option is evidenced by the still elevated fumarate production from glutamine (Figure 3) and the mildly higher level of G6PDH in UOK262WT cells compared with the human renal cortical epithelial (HRCE) cells (Figure 5), although still significantly lower than that of the FH null cells.

Since the oxidative branch of the PPP is an efficient means for producing cytoplasmic NADPH (two NADPH per ribose generated) in addition to supplying ribose, its high activity in FH null cells suggests that it is a major source of reducing power for biosynthetic purposes. High NADPH production would be necessary for the increased level of fatty acid synthesis, a NADPH dependent process, and for production of the fatty acid precursor, citrate, via the reductive carboxylation of glutamine derived alpha-ketoglutarate [21]. Reductive carboxylation can occur either in the cytoplasm or the mitochondria using respectively isocitrate dehydrogenase 1 and 2 (IDH1 and IDH2), both of which require NADPH to enzymically act in that direction [21]. As such, NADPH produced via the oxidative PPP can help fulfill the demand of cytoplasmic reductive carboxylation and fatty acid synthesis.

Our data shows that, as predicted on the basis of previous work demonstrating down-regulation of AMPK and the correlated up-regulation of acetyl CoA carboxylase, synthesis of several major fatty acids are indeed increased [20]. The loss of FH is associated with both the down-regulation of AMPK and TP53 [20], which are central to cancer cell metabolism in proliferative and survival pathways. TP53 has also been shown to be able to negatively regulate usage of the oxidative branch of the PPP, and thus down-regulation of TP53 could contribute to the increased activity [43].

Furthermore, recent studies have highlighted the importance of counteracting the increased levels of oxidative stress present in FH null cells. The activation of the Nrf2 transcription factor via the inactivating succination of its repressor, KEAP1, results in the up regulation of numerous genes involved in combating oxidative stress and increased levels of ROS [25–27,42]. An essential component of combating ROS is the availability of the NADPH and thus the up regulation of its production by the oxidative branch of the PPP in these cells would work in tandem with activated Nrf2 to protect the cells from critical levels of oxidative stress.

The combination of dysregulation in these metabolic regulators and the truncated Krebs cycle may underlie the metabolic reprogramming in the FH null cancer cells, which is distinct from the metabolic reprogramming of other cancer cell types. This includes the reliance on glutamine for lipid biosynthesis via reductive carboxylation [21], up regulation of the heme oxygenase pathway [22] and the up regulation of anti-oxidative stress pathways [24,25]. In the present study we show that the reprogramming in FH null cells is more extensive, and includes increased ribose production for rapid proliferation and enhancement of the oxidative pentose phosphate pathway for the generation of NADPH required for anabolism and essential for combating oxidative stress. Analysis of existing gene expression data demonstrates that the tumor model well represents the actual HLRCC tumors in regard to ribose production and that it was a common feature of HLRCC tumors. By understanding the critical metabolic reprogramming induced by the genetic defect of HLRCC, specific metabolic targets may be developed for the development of effective forms of therapy of this disease.

## Materials and Methods

### Ethics Statement

All human subjects work was approved by NCI Institutional Review Board Committee. Renal tumor and retroperitoneal lymph node from separate patients bearing HLRCC were removed surgically at Urologic Oncology Branch, National Cancer Institute, NIH. Written informed consent was obtained from all subjects. Patients were evaluated on approved NCI-IRB protocol 97-C-0147 and/or 03-C-0066.

### Tumor origins and cell culture

Tumor cell line UOK262 was derived from a metastatic retroperitoneal lymph node as characterized previously [18]. The cell line UOK268 was isolated from a separate individual’s primary renal lesion [19]. All cell lines were maintained in high glucose DMEM medium as previously reported [18]. UOK262WT and UOK262EV are isogenic cell lines derived from UOK262 into which either a copy of the wild type fumarate hydratase gene (WT) or an empty vector (EV) has been reintroduced as previously described [20].

### Cell culturing in tracers

[1,2- ^13^C_2_] D-glucose (>99% purity and 99% isotope enrichment for each carbon position - Isotec, Inc., Miamisburg, OH) mixed in 1:1 ratio with naturally occurring ^12^C glucose was provided by SidMap, LLC (Los Angeles, CA). All other tracers were purchased from Sigma-Aldrich-Isotec Co. (St. Louis, MO). Cells (5x10^4^) were seeded in triplicates into T75 cm^2^ flasks or 10-cm dish and allowed to grow overnight, rinsed with 1 x PBS (without calcium and magnesium) before tracer introduction. For the [1,2- ^13^C_2_]-glucose experiments, cells were incubated in 1:1 ^13^C tracer and unlabeled glucose at a final total glucose concentration of 25 mM in Dulbecco’s Modified Eagle’s Medium (DMEM) containing 4 mM L-glutamine, 10% heat-inactivated fetal bovine serum (FBS) (Sigma-Aldrich Co.) and 100 U x mL^-1^ penicillin and 100µg x mL^-1^ streptomycin (Invitrogen, Grand Island, NY). Experiments with [U-^13^C]-glucose as the tracer were carried out at 10 mM and those with [U-^13^C,^15^N]-glutamine (Sigma-Aldrich-Isotec Co.) as the tracer at 4 mM.

### Measurements of oxygen consumption and extracellular acidification

The XF96 extracellular flux analyzer (Seahorse Bioscience, North Billerica, MA) was used to measure oxygen consumption rate (OCR) and extracellular acidification rate (ECAR) [37]. Cells were plated at a density of 10,000 cells/well and incubated in a 37 °C/10% CO_2_ incubator for 24 hours in XF96 cell culture plate. Prior to the XF measurement, growth medium was exchanged with XF assay medium (DMEM with 25 mM glucose, 4 mM L-glutamine and 1 mM pyruvate but no bicarbonate and low phosphate). Cells were harvest and counted after the assays when needed. The OCR and ECAR were then adjusted to per 10000 cells for comparison between UOK262 and UOK262WT. The algorithm used to calculate OCR is Ksv, which allows more accurate analysis of OCR at very low values, as oppose to the standard Akos algorithm [44]. Mitochondrial inhibitors oligomycin and antimycin (Sigma, St. Louis MO) was used to measure ATP-coupled and total mitochondrial respiration, respectively, while the residual respiration from antimycin inhibition construed non-mitochondrial respiration.

### Quenching and cell extract preparation

Briefly, following incubation with [1,2- ^13^C_2_]-glucose for 48 hours, cells were trypsinized with 0.25% EDTA/Trypsin (Invitrogen) centrifuged (1,400 rpm, 4° C), washed twice in 1 x PBS, snap frozen in liquid nitrogen and stored at -80 °C. Metabolite extractions and analyses were performed as previously described [40] and below. Lactate was extracted from 100 µl cell culture media by ethylene chloride after acidification with HCl, derivatized to its propylamine-heptafluorobutyrate ester form before GC-MS analysis with chemical ionization detection. The *m/z* 328 to 331 (^13^C_1_ to ^13^C_3_-lactate) were monitored for ^13^C mass isotopologue determination. For the [U-^13^C]-glucose and [U-^13^C,^15^N]-glutamine experiments, cells on 10-cm plates were washed 3 times in cold PBS, quenched in cold acetonitrile, followed by extraction in acetonitrile: H_2_O:CHCl_3_ (2:1.5:1) and silylated in MTBSTFA for GC-MS analysis as described previously [5,30]. Media samples (100 µl) were extracted with cold 10% trichloroacetic acid as described previously [4].

### RNA ribose isotopologue analysis

RNA ribose was isolated by acid hydrolysis of cellular RNA after Trizol purification of cell extracts as previously described [40]. Ribose was hydrolyzed from RNA and derivatized to its aldonitrile acetate form using hydroxylamine in pyridine with acetic anhydride (Supelco) before GC-MS analyses with electron impact ionization. The ion clusters around the *m/z* 217 were monitored (carbons 3 to 5 of ribose) and *m/z* 242 (carbons 1 to 4 of ribose) [45] to determine concentration and fractional distribution of ^13^C in ribose.

### Gas chromatography/mass spectrometry (GC/MS)

For the [1,2- ^13^C_2_]-glucose experiments, mass spectral data for glucose, ribose, and lactate were obtained on an HP5973 mass selective detector connected to an HP6890 gas chromatograph. The GC-MS settings were as follows: GC inlet, 230 °C; transfer line, 280 °C; MS source, 230 °C; MS Quad, 150 °C. An HP-5 capillary column (30 m length, 250 µm diameter, 0.25 µm film thickness) was used for metabolite separation n. For the [U-^13^C]-glucose experiment, a Polaris GC-ion trap MS system (ThermoFinnigan, Austin, TX) was used to analyze the Krebs cycle metabolites with settings described previously [4].

### Data analysis and statistical methods from GC/MS

Tracer experiments were carried out using triplicates cultures for each cell model. Mass spectral analyses were carried out by three independent automatic injections of 1 µl samples by the automatic sampler and accepted only if the standard sample deviation was <1% of the normalized peak intensity. Statistical analysis was performed using the parametric unpaired, two-tailed independent sample *t* test with 99% confidence intervals, and *p* < 0.05 was considered to indicate significant differences in glucose carbon metabolism.

### NMR analysis of extracts

Extracts of cells and media from the [U-^13^C]-glucose or [U-^13^C,^15^N]-glutamine experiments were prepared for NMR as previously described [38,46,47]. NMR spectra were recorded at either 14.1 T or 18.8 T, 20 °C, using standardized parameters as described in appropriate figure legends and previously [38]. Positional isotopomers were quantified by peak integration, with correction for differential relaxation as described previously [35,48,49].

### Western blotting

Western blotting was performed as described previously [18] using either a Hexokinase 1 (HK1) antibody (#2024S, Cell Signaling Technologies) in 1:1000 dilution, a Lactate Dehydrogenase A (LDHA) antibody (#3582S, a Glucose 6-Phosphate Dehydrogenase (G6PDH) antibody (ab993, Abcam) in 1:500 dilution, Cell Signaling Technologies) in 1:1000 dilution, a Fatty Acid Synthase (FASN) antibody (sc-55580, Santa Cruz) in 1:1000 dilution or a β-Actin antibody (#A2522, Sigma) in 1:5000 dilution followed by blotting with the appropriate LI-COR secondary antibody at 1:10,000 dilution. Bands were visualized using a LI-COR Odyssey® imager and the LI-COR Odyssey® software.

### Analysis of Published Papillary and HLRCC Tumor Samples

The National Center for Biotechnology Information (NCBI) Geo DataSets database was queried for publically available datasets that included the term “HLRCC”. One query (GSE26574) was identified and contained CEL files for HLRCC (5) renal tumors, HLRCC-associated normal kidney samples (2), Papillary Type I renal tumors (22), Papillary type II renal tumors (12) and normal kidney samples (8) that had been analyzed on a similar gene expression array (Affymetrix Human Genome U133 Plus 2.0 Array) [42]. Data files were downloaded directly from Geo DataSets and uploaded into Partek Genomic Suite 6.6 (Partek Incorporated, St. Louis, MO). Robust multi-array Average (RMA) normalization was performed to normalize the data. We selected to assess gene expression of ribose sugar metabolism and Euclidean hierarchical clustering was performed for all samples using Partek Genomic Suite 6.6.

## Supporting Information

File S1 Includes supplementary methods, Table S1 and Figures S1-S6. Supplementary methods for the protein extraction for fumarate hydratase activity assay and for the fumarate hydratase enzyme assay protocol. Table S1, Fractional enrichment of intracellular metabolites derived from [U-^13^C]-glucose or [U-^13^C]-glutamine in FH null and restored cells. Figure S1, Fumarate Hydratase Enzyme Activity Assays. Figure S2, ^13^C enrichment in [U-^13^C]-glucose-derived nucleotide ribose and fumarate indicate that glucose is a minor carbon source for fumarate but a major carbon source for ribose in FH null and restored cells. Figure S3, ^13^C labelling patterns of intracellular lactate and alanine indicate the importance of glucose as their carbon source in UOK262 and UOK262WT cells. Figure S4, ^13^C labelling patterns of nucleotide Ribose in UOK262 cells indicate that glucose is the main source of carbon for ribose synthesis. Figure S5, GC/MS measurement for total ribose. Figure S6, Hierarchical clustering of Papillary renal tumors and normal kidney based on the expression levels of ribose sugar metabolism genes.
(DOCX)
