# Supplementary material for: Metabolic Reprogramming for Producing Energy and Reducing Power in Fumarate Hydratase Null Cells from Hereditary Leiomyomatosis Renal Cell Carcinoma
Source: PLoS One. 2013 Aug 15;8(8):e72179. doi: 10.1371/journal.pone.0072179 (PMC3744468; doi:10.1371/journal.pone.0072179)
Supplement: File S1 — Supplementary methods for the protein extraction for fumarate hydratase activity assay and for the fumarate hydratase enzyme assay protocol. Table S1, Fractional enrichment of intracellular metabolites derived from [U-13C]-glucose or [U-13C]-glutamine in FH null and restored cells. Figure S1, Fumarate Hydratase Enzyme Activity Assays. Figure S2, 13C enrichment in [U-13C]-glucose-derived nucleotide ribose and fumarate indicate that glucose is a minor carbon source for fumarate but a major carbon source for ribose in FH null and restored cells. Figure S3, 13C labelling patterns of intracellular lactate and alanine indicate the importance of glucose as their carbon source in UOK262 and UOK262WT cells. Figure S4, 13C labelling patterns of nucleotide Ribose in UOK262 cells indicate that glucose is the main source of carbon for ribose synthesis. Figure S5, GC/MS measurement for total ribose. Figure S6, Hierarchical clustering of Papillary renal tumors and normal kidney based on the expression levels of ribose sugar metabolism genes. (DOCX) [file pone.0072179.s001.docx]

**Supporting Information : File S1**

**Metabolic Reprogramming for Producing Energy and Reducing Power in Fumarate Hydratase Null Cells from Hereditary Leiomyomatosis Renal Cell Carcinoma**

Youfeng Yang ^1*^, Andrew N. Lane ^2,3*^,Christopher J. Ricketts ^1^, Carole Sourbier ^1^, Ming-Hui Wei ^1^, Brian Shuch ^1^, Lisa Pike ^4^, Min Wu ^4,5^, Tracey A. Rouault ^6^, Laszlo G. Boros ^7,8^, Teresa W-M. Fan ^2,3,9^, W. Marston Linehan ^1^

Affiliations:

^1^ Urologic Oncology Branch, Center for Cancer Research, National Cancer Institute, National Institutes of Health, Bethesda, MD, USA.

^2^ J.G. Brown Cancer Center, University of Louisville, Louisville, KY, USA.

^3^ Center for Regulatory and Environmental Analytical Metabolomics (CREAM), University of Louisville, Louisville, KY, USA.

^4^ Seahorse Bioscience, North Billerica, MA, USA.

^5^ Center for Human Genetic Research, Richard B. Simches Research Center, Massachusetts General Hospital, Boston, MA, USA. (current address)

^6^ Molecular Medicine Program, *Eunice Kennedy Shriver* National Institutes of Child Health and Development, Bethesda, MD, USA.

^7^ SIDMAP LLC (2990 South Sepulveda Blvd.), Los Angeles, CA, USA.

^8^ UCLA School of Medicine, Los Angeles, CA, USA.

^9^ Department of Chemistry, University of Louisville, Louisville, KY, USA.

*These authors shared equally in this work.

Correspondence to:

W. Marston Linehan, M.D.

Urologic Oncology Branch, National Cancer Institute, 10 Center Drive MSC 1107, Building 10 CRC Room 1-5940, Bethesda, MD 20892-1107, USA

Tel: 301-496-6353

Fax: 301-402-0922

Email: [WML@nih.gov](mailto:WML@nih.gov)

and

Teresa W.-M. Fan, Ph.D.

University of Louisville

Center for Regulatory & Environmental Analytical Metabolomics (CREAM)

2210 S. Brook St., Rm 335 John W. Shumaker Research Bldg

Louisville, KY 40208

Office (502) 852-6448

Email: twmfan@gmail.com

**Supplementary Methods**

*Fumarate Hydratase Enzyme Assay* - The Fumarate Hydratase enzyme assay was performed as described in Yang *et al* 2010^1^ and is described below. All reagents were purchased from Sigma-Aldrich Co. (St. Louis, MO).

*Protein Extraction for Fumarate Hydratase Activity Assay*

For total protein lysates, cells were trypsinized, pelleted following washes with PBS and re-suspended in 1ml of 50 mM HEPES-KOH pH7.6 solution. Once re-suspended the cell pellet suspension was sonicated twice for 5-10 seconds each time and chilled in between each sonication. The suspension was then centrifuged at 900g at 4°C for 8 minutes and the supernatant transferred to an appropriately labeled fresh centrifuge tube before being assayed for protein concentration.

For mitochondrial protein lysates, cells were trypsinized, pelleted following washes with PBS and re-suspended in 1ml of 0.25M Sucrose/25 mM HEPES-KOH pH7.6 solution. Once re-suspended the cell pellet suspension was sonicated twice for 5-10 seconds each time and chilled in between each sonication. The suspension was centrifuged at 900g at 4°C for 8 minutes, and then 700ul of the supernatant was transferred to a fresh centrifuge tube and centrifuged at 14,000g at 4°C for 10 minutes. The supernatant was discarded and the remaining pellet washed with 700ul of 0.25M Sucrose/25 mM HEPES-KOH pH7.6 solution before being centrifuged at 14,000g at 4°C for 10 minutes. Once all the supernatant was again discarded the remaining pellet was suspended in 100ul of chilled 50 mM HEPES-KOH pH7.6 solution before being assayed for protein concentration.

*Fumarate Hydratase Enzyme Assay Protocol*

1. For each sample to be assayed the following reagents were mixed in a 1.5ml centrifuge tube:

826ul 50 mM HEPES-KOH pH7.6 solution

4ul 1M MgCl solution

5ul 1M KPO_4_ monobasic solution

1. The reagents were then mixed and pre-warmed to 30°C, as was 500ul of Fumaric acid solution (20 mg/ml0.
2. Before reading each sample on the Spectrophotometer the following reagents were added to the 1.5ml centrifuge tube after which it was returned to the 30°C hot block for 3-5 minutes:

15ul NAPD (20mg/ml) solution. (Freshly prepared)

40ul NADP Malic enzyme solution. (Freshly prepared)

50ul 5mg of total/mitochondrial protein lysate plus the relevant amount of distilled water.

1. Once re-warmed to 30°C, 60ul of fumaric acid solution was added to the centrifuge tube; the contents were well mixed and then placed in a 1ml cuvette before being immediately placed in a spectrophotometer.
2. The absorbance at a wavelength of 340nm was then measured and recorded every 30 seconds using the enzyme kinetic mode on the DU 530 Life Science UV/Vis Spectrophotometer (Beckman Coulter, Inc.).
3. Three samples were measured for each line cell and the average activities for each cell line were compared to a control cell line.

1. Yang Y, Valera VA, Padilla-Nash HM, Sourbier C, Vocke CD, et al.: UOK 262 cell line, fumarate hydratase deficient (FH-/FH-) hereditary leiomyomatosis renal cell carcinoma: in vitro and in vivo model of an aberrant energy metabolic pathway in human cancer. Cancer Genet Cytogenet, 196**:** 45, 2010.

**Table S1: Fractional enrichment of intracellular metabolites derived from [U-^13^C]-glucose or [U-^13^C]-glutamine in FH null and restored cells**

UOK262 (FH null) and UOK262WT (FH restored) cells were grown for 24 h in media containing either [U-^13^C]-glucose or [U^13^C,^15^N]-glutamine as described in Methods. Fractional ^13^C enrichment in cellular metabolites was determined from 1D or 2D NMR analysis (cf. Figs. S1-3), as described in Methods.

| Compound | UOK262 | UOK262WT |
| --- | --- | --- |
| %^13^C Lac from ^13^C Glc | 95 | 93 |
| %^13^C Lac from ^13^C Gln | nd^a^ | nd^a^ |
| %^13^C fumarate from ^13^C Gln | 88% | 80 % |
| ^13^C fumarate/ATP^b^ | 0.6 | 0.08 |
| %^13^C Uri in UXP from ^13^C Gln | 25 | 35 |
| %^13^C Uri in UXP from ^13^C Glc | <5 | 8 |
| %^13^C ribose from ^13^C Glc | >95 | >95 |
| ^13^C ribose from ^13^C Gln | NA^c^ | NA^c^ |

^a^ nd : not detected (<2% enrichment)

b ratio of fumarate to ATP concentration by NMR

^c^ NA : natural ^13^C abundance (1.1 %)

**Figure S1: Fumarate Hydratase Enzyme Activity Assays.**

Total protein lysates, and selected mitochondrial protein lysates, were extracted from cell lines as described in the supplementary methods. The cell lines analyzed included UOK262, UOK262EV and UOK262WT with HRCE and HEK-293 cell lines used to represent normal control cells. These lysates were analysed *in-vitro* for fumarase enzyme activity, as described in the supplementary methods, and measurements of absorbance at 340nm were taken every 30 seconds for 10 minutes.

1. The absorbance at 340nm for each cell time every 30 seconds for 10 minutes.
2. The fumarate hydratase activity of the total protein lysates, measured by absorbance at 340nm after 10 minutes, is shown for all the cell lines as a percentage of the HRCE control cell line’s activity. Bars represent the average of three experiments and the error bars represent the standard deviation.
3. The fumarate hydratase activity of the selected mitochondrial protein lysates, measured by absorbance at 340nm after 10 minutes, is as a percentage of the HEK-293 control cell line’s activity. Bars represent the average of three experiments and the error bars represent the standard deviation.

**Figure S2: ^13^C enrichment in [U-^13^C]-glucose-derived nucleotide ribose and fumarate indicate that glucose is a minor carbon source for fumarate but a major carbon source for ribose in FH null and restored cells.**

Both UOK262 and UOK262WT cells were cultured in 10 mM [U-^13^C]-glucose + 4 mM unlabelled glutamine for 24 h. 1D ^1^H NMR spectra of both cell (A) and medium (B) extracts were recorded at 800 MHz, 20 °C with an acquisition time of 2 s and a recycle time of 5 s. The data were processed with a 1 Hz line broadening exponential function. The near absence of ^13^C satellite in the fumarate peak at 6.55 ppm in panel A indicates that little glucose was metabolized into fumarate. In contrast, the ^13^C satellites of AXP at 6 and 6.24 ppm was prominent, indicating that glucose is a major carbon source for the synthesis of nucleotide ribose. Likewise, glucose is the major precursor for lactate released into the medium by both cell types, as shown by the high abundance of the ^13^C_3_-lactate resonances in the medium spectra (B). Also shown is the greater production and release of ^13^C_3_-lactate by UOK262 than UOK262WT cells.

**Figure S3: ^13^C labelling patterns of intracellular lactate and alanine indicate the importance of glucose as their carbon source in UOK262 and UOK262WT cells.**

Cells were grown in the presence of [U-^13^C]-glucose or [U-^13^C]-glutamine for 24 h before extraction as described in Materials and Methods. The 2D ^1^H TOCSY spectra of UOK262 cell extracts (A,C) were recorded at 800 MHz, 20°C with acquisition times of 0.3 s in t_2_ and 0.035 s in t_1_, and with a 50 ms spin lock at 8 kHz strength. The data were processed using an unshifted gaussian and a 1 Hz line broadening in both dimensions. The data in t_1_ were linear predicted and zerofilled once prior to Fourier transformation. The 1D ^1^H spectra (B,D) were acquired and processed as described in Fig. S1. Black and red spectra in panels B and D represent UOK262WT and UOK262 cells, respectively.

1. [U-^13^C]-glucose as tracer. Rectangular boxes in the TOCSY panel (A) delineate the ^13^C satellites of the H2-H3 cross peaks of lactate and alanine, which show a uniform labelling pattern. The corresponding ^13^C satellite patterns in the overlaid 1D spectra of both cells indicate uniform labelling in all three carbons of lactate and alanine ([1](#_ENREF_1)), i.e. both metabolites were derived from glucose without scrambling. The black dashed line depicts the covalent connectivity from the ^12^C attached H2 to H3 of threonine. No ^13^C satellites of H4-H2 or H4-H3 were observed for this essential amino acid, which was derived from the medium.
2. [U-^13^C]-glutamine as tracer. Similar to threonine, no satellites were detectable in lactate or alanine, indicating that Gln is not a significant source of pyruvate in UOK262 and UOK262WT cells.

**Figure S4: ^13^C labelling patterns of nucleotide Ribose in UOK262 cells indicate that glucose is the main source of carbon for ribose synthesis.**

Cells were grown in the presence of [U-^13^C]-glucose or [U-^13^C]-glutamine for 24 h before extraction, as described in Materials and Methods. The TOCSY spectra were recorded and processed as described in Fig. S2.

1. [U-^13^C]-glucose as tracer. Rectangular boxes delineate the ^13^C satellites of the H1’-H2’ cross peak in adenine (AXP), uracil (UXP), and cytosine nucleotides (CXP), as well as the ^13^C satellites of the H1’-H2’ cross peaks of adenine (A1’→A2’) and nicotinamide (N1’→N2’) rings in NAD^+^.
2. [U-^13^C]-glutamine as tracer. No ^13^C satellites were apparent for any nucleotide.

Thus, glucose is the major carbon source of nucleotide ribose biosynthesis in these cells.

**Figure S5: GC/MS measurement for total ribose.**

The relative levels of ribose sugars were determined by Gas chromatography/mass spectrometry (GC/MS) of UOK262, UOK262EV, UOK268 and UOK262WT and presented as a percentage of the UOK262WT. The bar graph shows the mean values of three replicates along with the standard deviation as error bars. * indicates a statistically significant result (p<0.05) from a T-Test.

**Figure S6: Hierarchical clustering of Papillary renal tumors and normal kidney based on the expression levels of ribose sugar metabolism genes.**

The Affymetrix Human Genome U133 Plus 2.0 Array expression data for 5 HLRCC renal cancer samples (red), 2 HLRCC-associated normal kidney samples (yellow), 22 Papillary Type I renal cancer samples, 12 Papillary Type II renal cancer samples and 8 normal kidney samples (green) [42] was clustered using Euclidean hierarchical clustering (Partek Genomic Suite 6.6) based on the expression data for selected ribose sugar metabolism genes. The heat map was shaded to indicate comparative over-expression in red and under-expression in green.

TKT: Transketolase, TALDO1 Transaldolase 1, RPE: Ribulose-5-Phosphate-3-Epimerase, G6PD: Glucose-6-Phosphate Dehydrogenase, PGLS: 6-Phospho-gluconolactonase, RPIA: Ribose 5-Phosphate Isomerase A, PGD: Phosphogluconate Dehydrogenase, TKTL1: Transketolase-like 1, TKTL2: Transketolase-like 2.

1. Fan TW-M, Kucia M., Jankowski K, Higashi RM, Rataczjak MZ, Rataczjak J *et al*. Proliferating Rhabdomyosarcoma cells shows an energy producing anabolic metabolic phenotype compared with Primary Myocytes. 2008 *Molecular Cancer* **7:** 79.


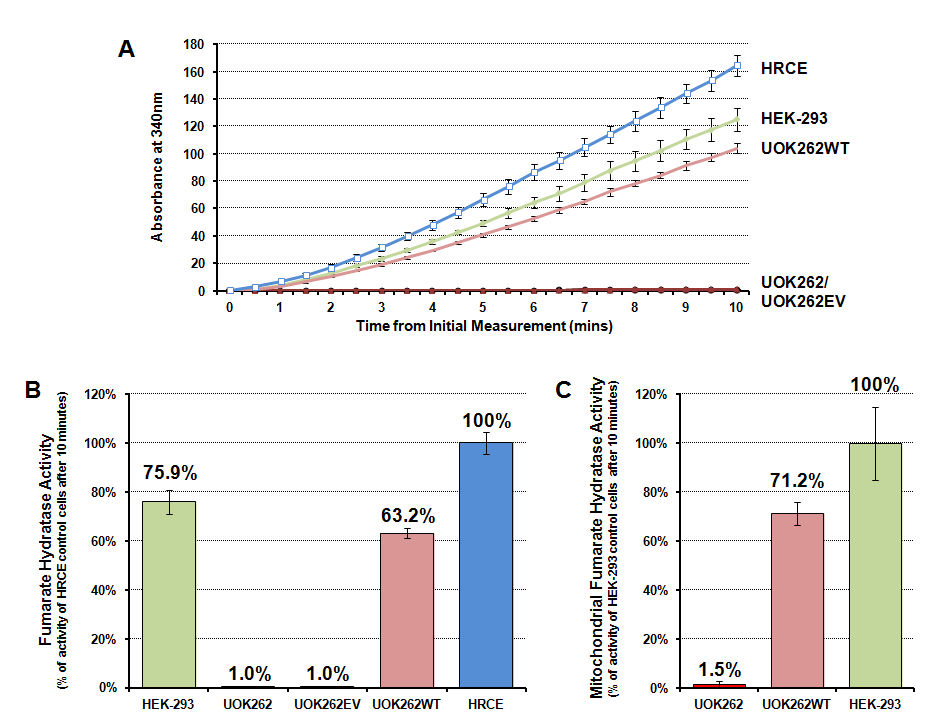


**Figure S1**

**Figure S2**


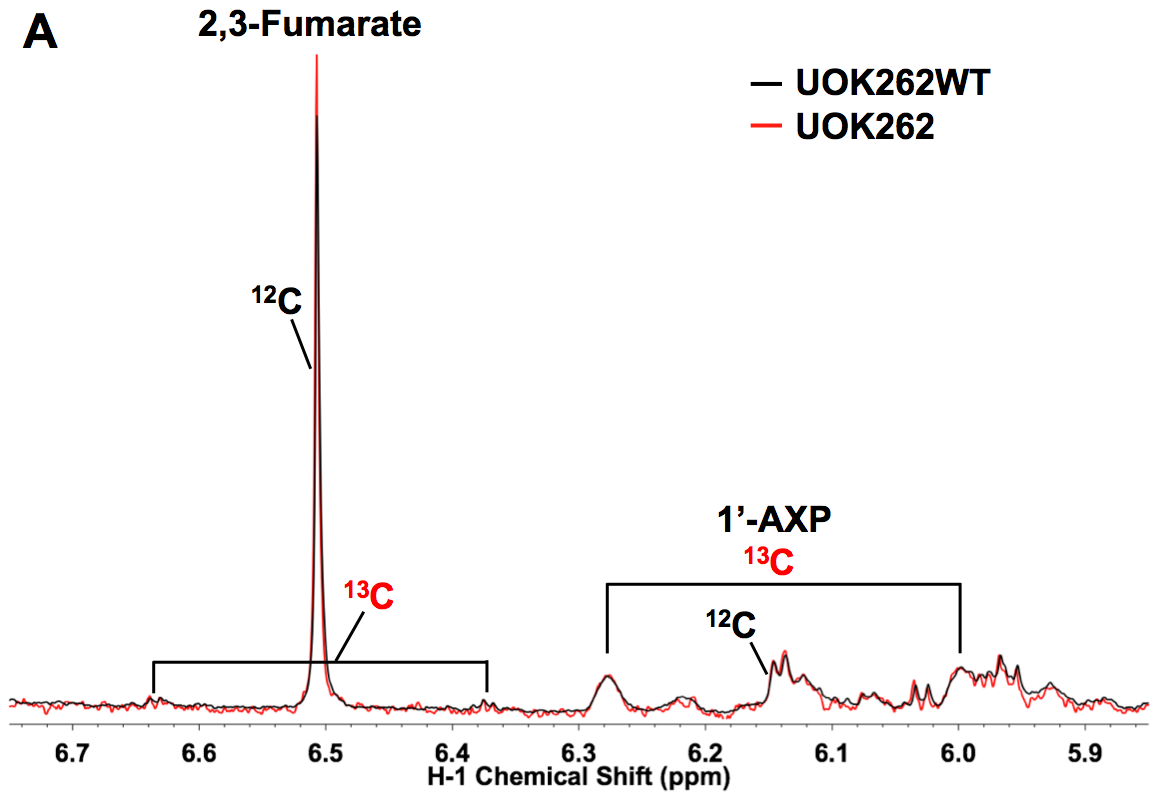


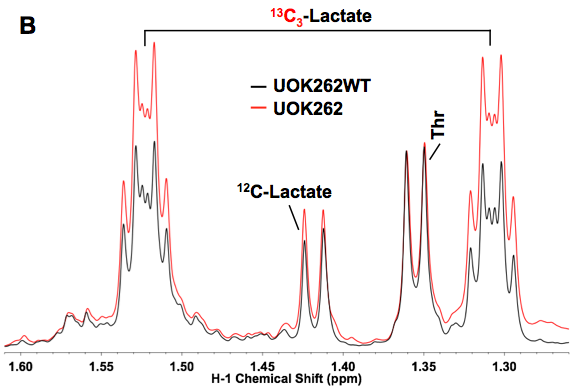


**Figure S3**


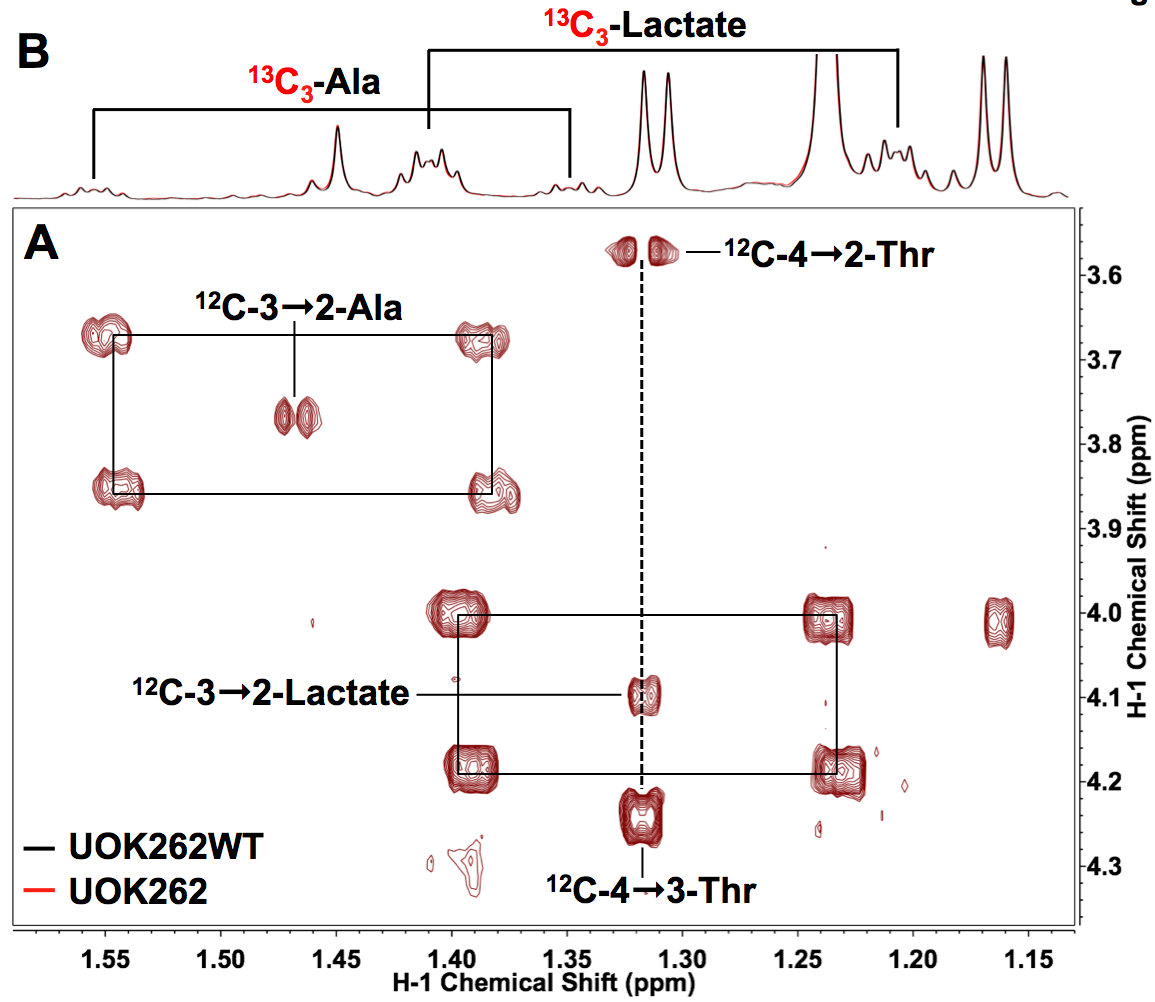


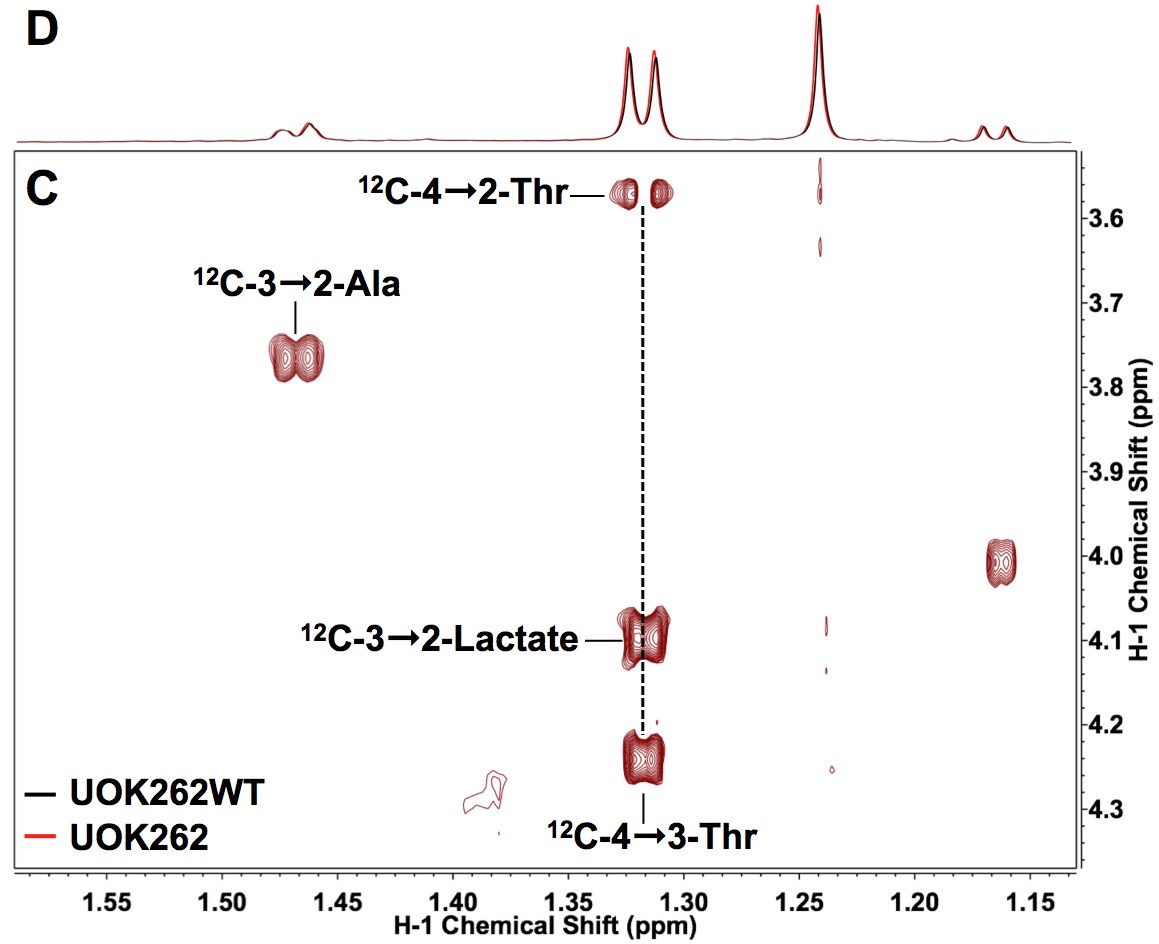


**Figure S4**


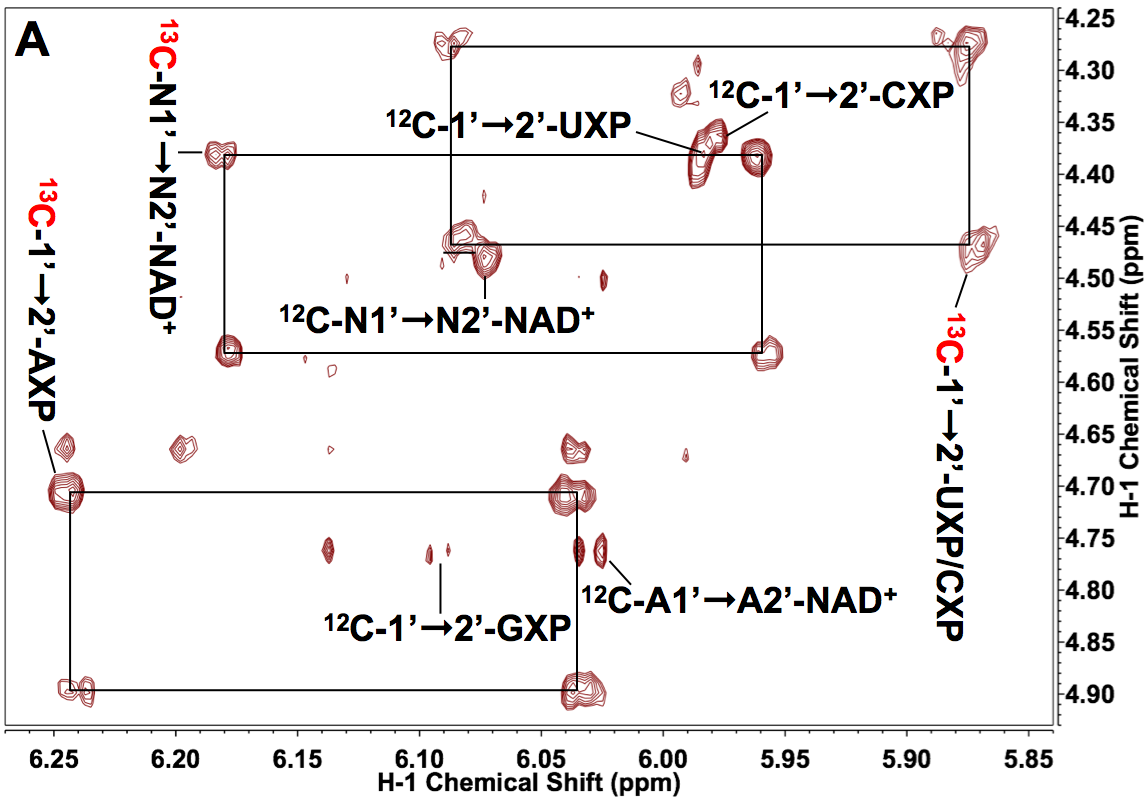

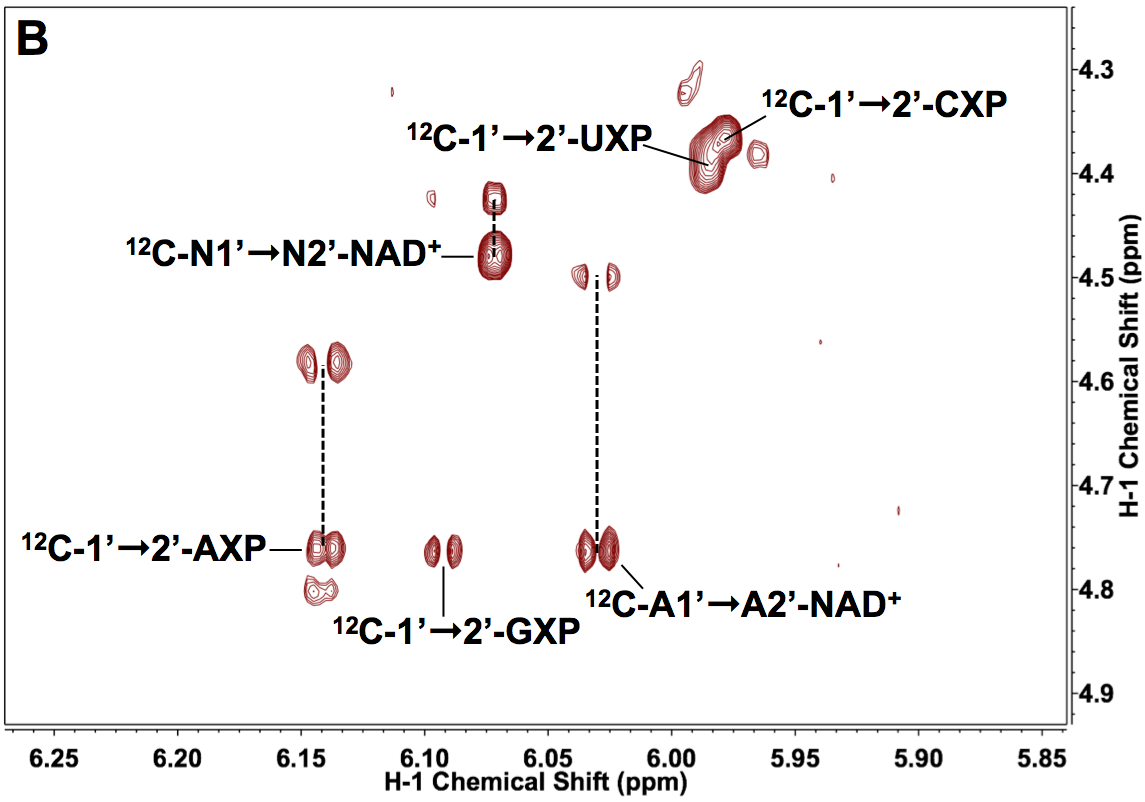


**Figure S5**


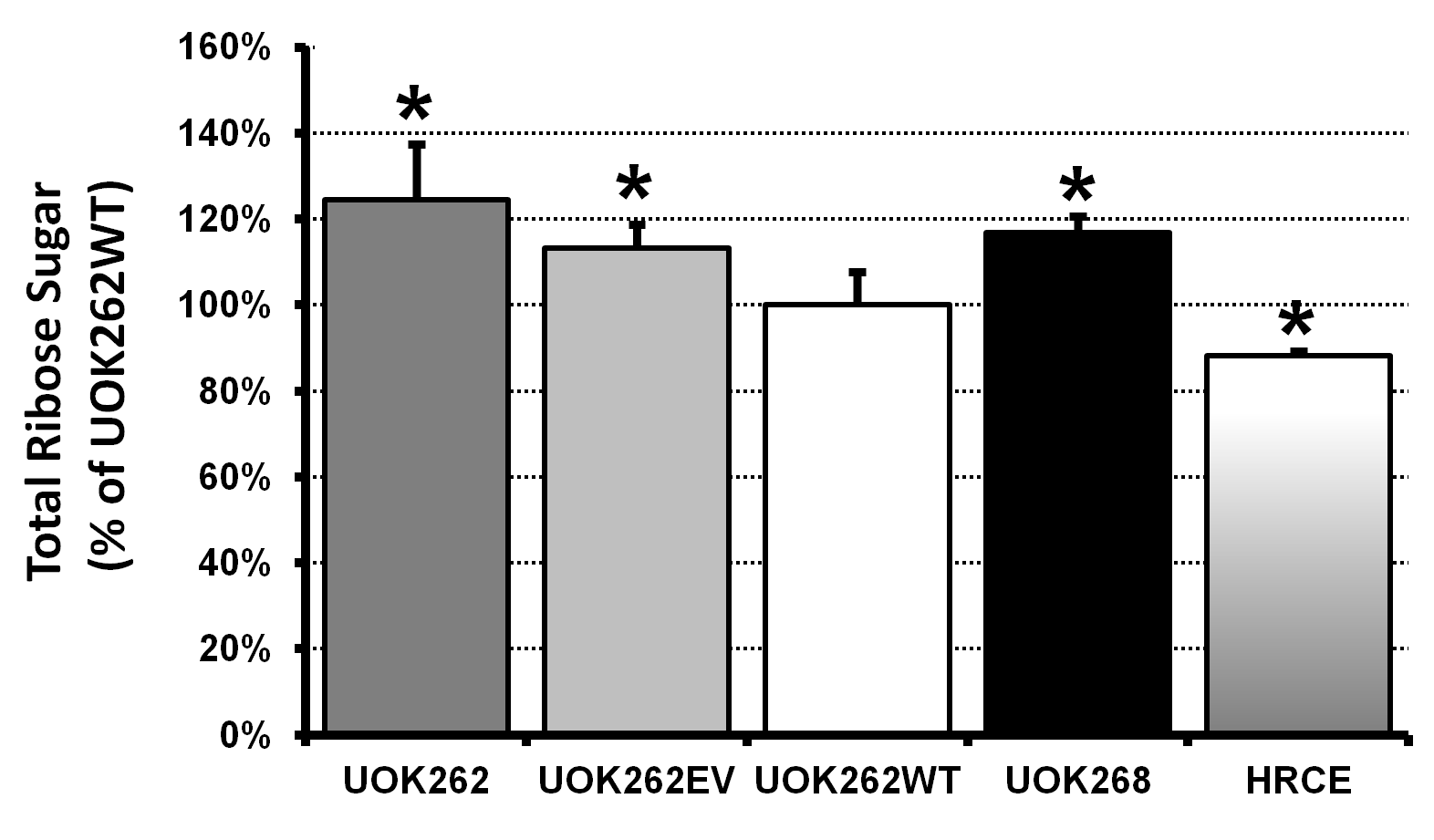


**Figure S6**
